# Partial reprogramming strategy for intervertebral disc rejuvenation by activating energy switch

**DOI:** 10.1111/acel.13577

**Published:** 2022-03-09

**Authors:** Feng Cheng, Chenggui Wang, Yufei Ji, Biao Yang, Jiawei Shu, Kesi Shi, Lulu Wang, Shaoke Wang, Yuang Zhang, Xianpeng Huang, Xiaopeng Zhou, Kaishun Xia, Chengzhen Liang, Qixin Chen, Fangcai Li

**Affiliations:** ^1^ Department of Orthopedics Surgery The Second Affiliated Hospital School of Medicine Zhejiang University Hangzhou China; ^2^ Orthopedics Research Institute of Zhejiang University Hangzhou China; ^3^ Key Laboratory of Motor System Disease Research and Precision Therapy of Zhejiang Province Hangzhou China; ^4^ Department of Orthopedics The Second Affiliated Hospital and Yuying Children’s Hospital of Wenzhou Medical University Wenzhou China; ^5^ Department of Gastrointestinal Surgery Xiamen Cancer Center The First Affiliated Hospital of Xiamen University Xiamen China; ^6^ Laboratory of Metabolism and Cell Fate Guangzhou Institutes of Biomedicine and Health Chinese Academy of Sciences Guangzhou China

**Keywords:** cellular senescence, intervertebral disc degeneration, nucleus pulposus cells, reprogramming

## Abstract

Rejuvenation of nucleus pulposus cells (NPCs) in degenerative discs can reverse intervertebral disc degeneration (IDD). Partial reprogramming is used to rejuvenate aging cells and ameliorate progression of aging tissue to avoiding formation of tumors by classical reprogramming. Understanding the effects and potential mechanisms of partial reprogramming in degenerative discs provides insights for development of new therapies for IDD treatment. The findings of the present study show that partial reprogramming through short‐term cyclic expression of Oct‐3/4, Sox2, Klf4, and c‐Myc (OSKM) inhibits progression of IDD, and significantly reduces senescence related phenotypes in aging NPCs. Mechanistically, short‐term induction of OSKM in aging NPCs activates energy metabolism as a “energy switch” by upregulating expression of Hexokinase 2 (HK2) ultimately promoting redistribution of cytoskeleton and restoring the aging state in aging NPCs. These findings indicate that partial reprogramming through short‐term induction of OSKM has high therapeutic potential in the treatment of IDD.

## Graphical abstract

Partial reprogramming through short‐term cyclic expression of Oct‐3/4, Sox2, Klf4, and c‐Myc (OSKM) inhibits the progression of intervertebral disc degeneration. Mechanistically, short‐term induction of OSKM in aging nucleus pulposus cells (NPCs) activates energy metabolism as a “energy switch” by upregulating expression of Hexokinase 2 (HK2) ultimately promoting redistribution of cytoskeleton and restoring the aging state in aging.

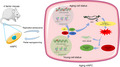

**Abbreviations**
3‐Br PA: 3‐bromopyruvic acid
4F: four factors
AF: annulus fibrosus
DG: dentate gyrus
dox: doxycycline
ECM: extracellular matrix
HDF: human dermal fibroblasts
HGPS: Hutchinson‐Gilford progeria syndrome
HK2: hexokinase 2
IDD/IVDD: intervertebral disc degeneration
IVD: intervertebral disc
LBP: low back pain
NP: nucleus pulposus
NPCs: nucleus pulposus cells
OSK: Oct4, Sox2 and Klf4
OSKM: Oct‐3/4, Sox2, Klf4 and c‐Myc
OxPhos: oxidativephosphorylation
ROS: reactive oxygen species
RS: replicative senescence
SASP: senescence‐associated secretory phenotype
TAC: tricarboxylic acid cycle

## 1 INTRODUCTION

Reprogramming of somatic cells by overexpression of Yamanaka factors (Oct‐3/4, Sox2, Klf4, and c‐Myc [OSKM]) can reverse cell differentiation and promote terminal differentiated cells to attain pluripotent state (Polo et al., 2012; Takahashi et al., 2007; Takahashi & Yamanaka, 2006). However, the process induces tumor formation *in vivo* (Abad et al., 2013; Ohnishi et al., 2014; Shibata et al., 2018). Partial reprogramming method (Lu et al., 2020; Rodríguez‐Matellán et al., 2020; Sarkar et al., 2020) through short‐term cyclic expression of reprogramming factors circumvents the limitation of tumor formation and can be applied in vivo (Ocampo et al., 2016). Partial reprogramming ameliorates cellular aging and mice aging state. Moreover, Hutchinson–Gilford progeria syndrome (HGPS) induced aging cell hallmarks such as nuclear envelope abnormalities, shorter telomere size, and increased oxidative stress are reversed by partial programming (Ocampo et al., 2016). In addition, transient reprogramming of nuclear factors resets epigenetic clock, reduces inflammation of chondrocytes, and restores the youthful regenerative response in human muscle stem cells (Sarkar et al., 2020). Furthermore, the short‐term induction of Oct4, Sox2, and Klf4 (OSK) promotes axon regeneration after injury, and reverses vision loss in an aged mouse model of glaucoma (Lu et al., 2020). Transient cyclic reprogramming in the dentate gyrus (DG) increases the survival of newborn DG neurons during their maturation and increases synaptic plasticity in mature neurons (Rodríguez‐Matellán et al., 2020). These findings indicate that partial reprogramming is a potential strategy for slowing aging.

Low back pain (LBP) is strongly associated with intervertebral disc degeneration (IDD) and a major cause of disability worldwide. LBP imposes an enormous clinical and socioeconomic burden on society (“Global, regional, and national mortality among young people aged 10–24 years, 1950–2019: a systematic analysis for the Global Burden of Disease Study 2019,” 2021). Intervertebral disc (IVD) combines with a gel‐like nucleus pulposus (NP) and circumferentially annulus fibrosus (AF) (Roberts et al., 2006). NP plays essential roles in maintaining homeostasis by secreting type II collagen and proteoglycans to form extracellular matrix (ECM) (Wang et al., 2013). Although, the precise pathogenesis of IDD remains elusive, recuperating NP aging state can arrest progression of IDD. IVD exhibits unique features such as avascularity, hypoxia, acidic environment, low nutrition, and low cellularity (Sakai & Grad, 2015). Therefore, glycolysis is the main energy metabolism pathway in NP cells (Madhu et al., 2020). Aging NP cells have low glycolysis levels resulting in severe energy stress and high levels of reactive oxygen species (ROS). Yamanaka factors have high potential in enhancing the process of glycolysis (Sone et al., 2017). Therefore, the present study sought to explore whether the upregulation of expression of Yamanaka factors in aging NP cells can ameliorate the energy stress and restore the young state. Moreover, that the role of partial reprogramming by Yamanaka factors in treatment of IDD was evaluated.

**SCHEME 1:**
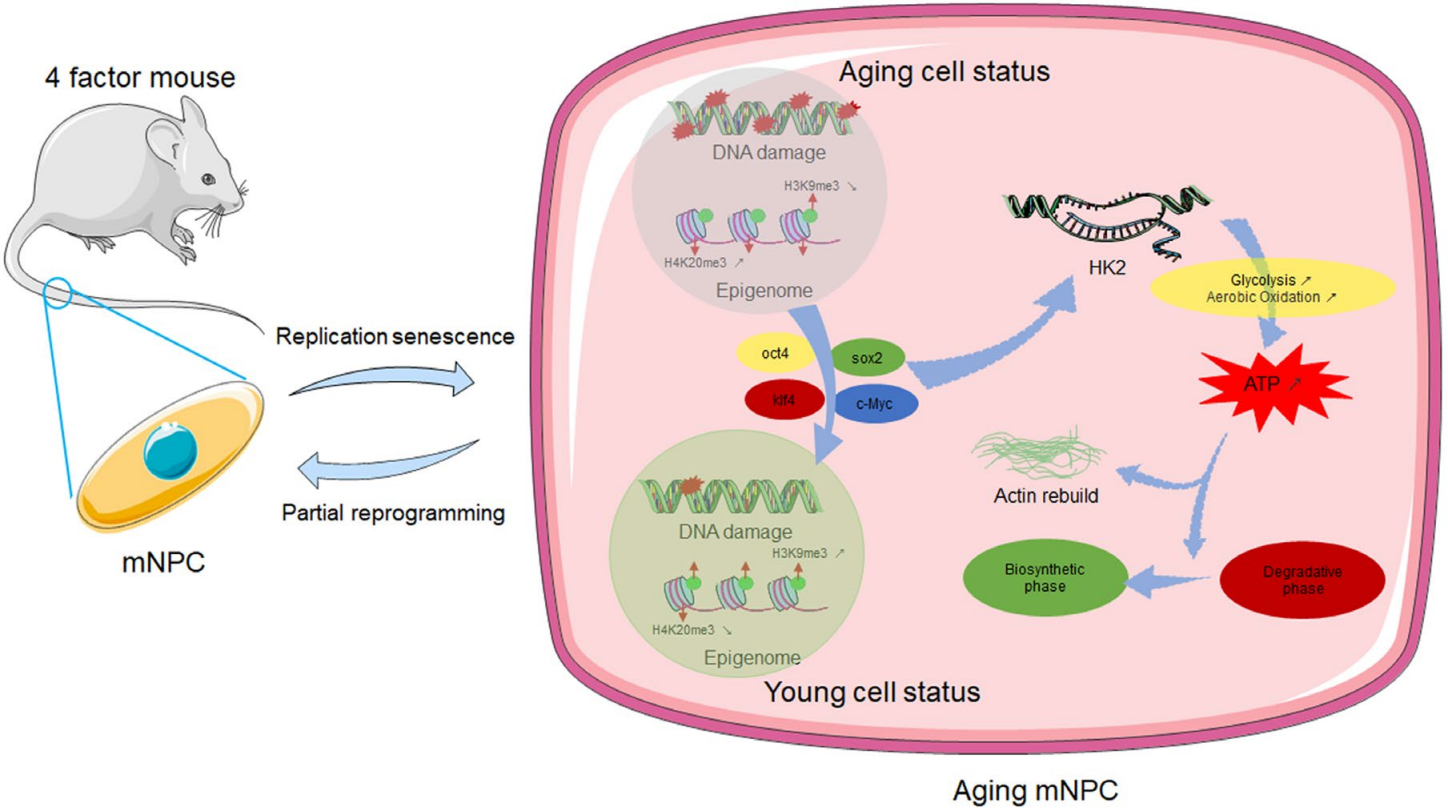
Schematic Diagram of partial reprogramming in aging NPCs

In the current study, surgically induced IDD mouse model was established to explore the effect of short‐term cyclic induction of OSKM in vivo. In addition, NP cells (NPCs) were isolated and cultured to P6 to establish a replicative senescence (RS) model of NPCs (aging NPCs). This model was used to explore the potential mechanism of amelioration of IDD in vitro. The findings showed that short‐term cyclic OSKM induction ameliorates progression of IDD. Partial reprogramming inhibits phenotypes associated with aging in aging NPCs by upregulating the expression of hexokinase 2 (HK2) to activate energy metabolism and promote redistribution of the cytoskeleton (Scheme 1). These changes induce a “younger” state in aging NPCs. These results indicate that partial reprogramming induced by OSKM improves aging state of NPCs and delays progression of IDD.

## 2 RESULTS

### 2.1 In vivo short‐term cyclic reprogramming ameliorates intervertebral disc degeneration

**FIGURE 1:**
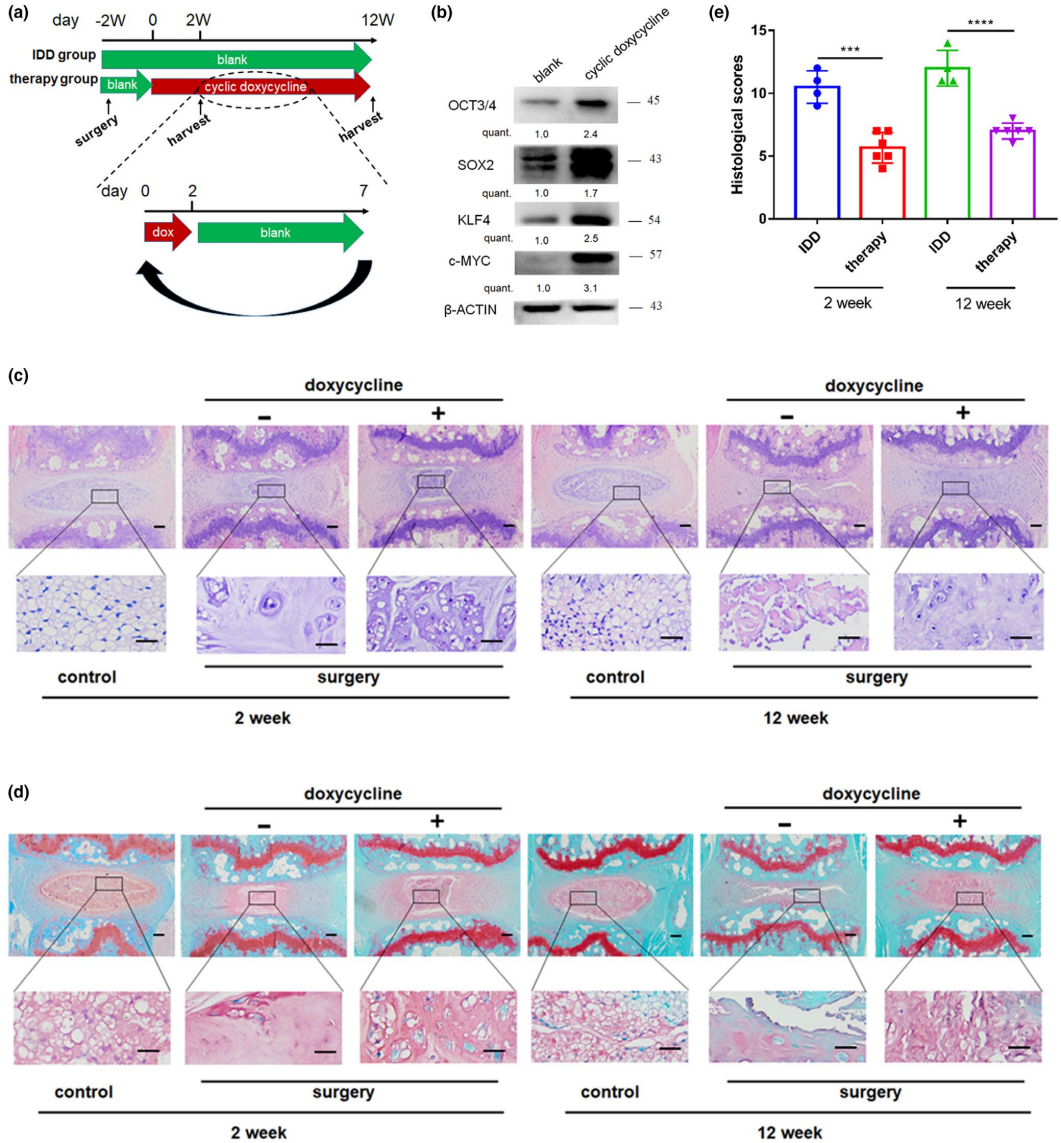
In vivo short‐term cyclic reprogramming ameliorates intervertebral disc degeneration. (a) Overview of the experimental set‐up on induction of doxycycline after surgery and representation of cyclic doxycycline administration protocol. (b) 4 factor mice were administered with doxycycline (1 mg/ml) through drinking water or not for 2 days and then the levels of Oct4, Sox2, Klf4, and c‐Myc from NP tissue were determined by Western blotting. (c,d) HE (c) and Safranin O (d) staining of disc sections in control groups and treatment groups at Week 4 and Week 14 after surgery and blank groups (water groups, *n* = 5; doxycycline groups, *n* = 5; blank groups, *n* = 5). Scale bar = 50 μm. (e) Histological score of mice in control groups and treatment groups mice, as determined by Safranin O staining. ***p < 0.001, ****p < 0.0001 unpaired two‐sample Student's *t*‐test

Metabolic disease and muscle injury in aging mice can be ameliorated by transient expression of Yamanaka factors in vivo (Ocampo et al., 2016); however, studies have not elucidated whether this kind of partial reprogramming in nucleus pulposus cells (NPCs) can promote degenerative disc regeneration. Therefore, the effect of cyclic OSKM induction in 4F mice from 8 weeks of age was explored in the present study and the genotype for each mouse was evaluated (Supplementary Figure S1a). Intervertebral disc degeneration (IDD) model was established by AF needle puncture after degeneration for 2 weeks. Doxycycline (dox) was administered for 2 days and then withdrawn for 5 days in the treatment group. The treatment groups received several cycles of the treatment regimen whereas the IDD group received normal water (Figure 1a). Results from qPCR (Supplementary Figure S1b–e) and WB (Figure 1b) showed specific expression of Oct4, Sox2, Klf4, and c‐Myc in disc tissue after administration of dox for 2 days. Mice in the control group presented well‐organized NP and annulus fibrosus (AF). Histological analysis at 4 weeks post‐surgery in mice administered with dox for 2 weeks showed slighter degenerative changes in IVDs compared with mice in the IDD group. This finding indicates significant different damage of NP and AF in IVDs. This phenotype was more evident at Week 14 post‐surgery. Mice in the treatment group which were induced short cycle of dox presented less damage of discs compared with mice in the IDD group after the same period (Figure 1c–d and Supplementary Figure S1f), These results were verified by significant decrease in histologic scores (Figure 1e). Further, three groups including 4F mice at 2 weeks of age (2 week group), 12 months of age (12 month group), and 12 months of age received dox cyclic treatment for 2 weeks (12 month@cyclic doxycycline) to explore the effect of age on IVD. Histological analysis showed that dox‐induced 2‐week‐old 4F mice presented less degenerative changes compared with the 12 month group (Supplementary Figure S1g–h). 5‐mC% is an indicator of the level of DNA methylation. Notably, DNA hypomethylation is directly proportional to cell aging (Murgatroyd et al., 2010). The findings showed that 5‐mC% significantly increased after dox cyclic treatment of IVD in aged 4F mice (Supplementary Figure S1i). Moreover, the expression of aging relative gene was improved at varying degrees (Supplementary Figure S1k–q). In summary, these findings indicate that short‐term cyclic reprogramming in 4F mice alleviates surgically induced IDD and age‐related IVD can be also reversed through cyclic OSKM induction.

### 2.2 Partial reprogramming rejuvenates phenotypes associated with aging in degenerative nucleus pulposus cells

**FIGURE 2:**
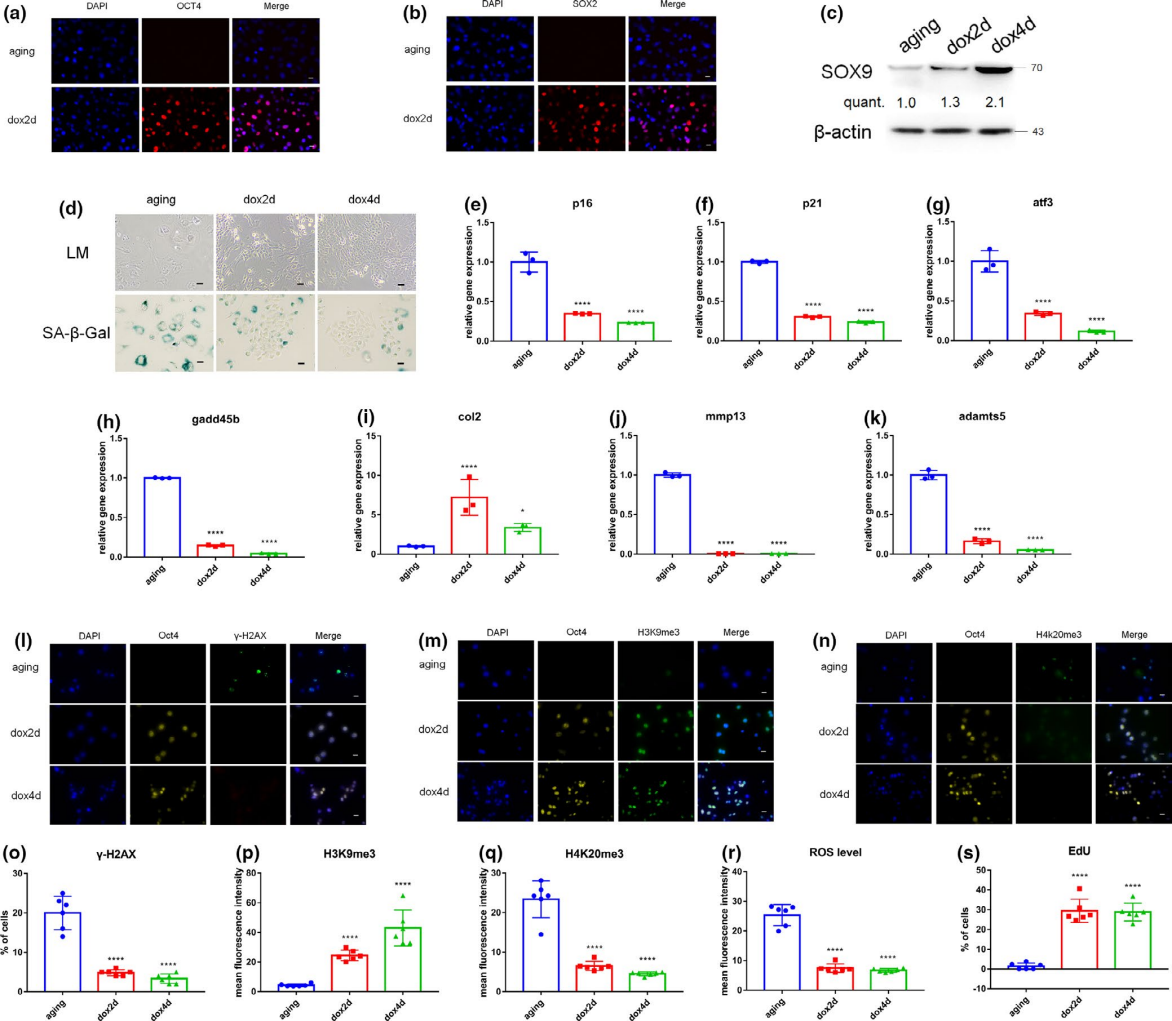
Partial reprogramming promotes phenotypes associated with aging in degenerative nucleus pulposus cells. (a,b) Immunofluorescence intensity of Oct4 (a) and Sox2 (b) in 4F aging NPCs after doxycycline treatment. Scale bar, 25 μm. (c) Cultured primary 4F aging NPCs were treated with doxycycline (1 μg/ml) for 2 days (dox2d) and 4 days (dox4d), then the levels of sox9 were determined by Western blotting. (d) Light microscope (LM) and β‐galactosidase activity in aging, dox2d, and dox4d samples. LM, scale bar, 100 μm; β‐galactosidase staining, scale bar, 50 μm. (e–h) qPCR analysis of stress response genes in the p53 pathway in 4F aging NPCs after doxycycline treatment. (i–k) qPCR analysis of senescence‐associated catabolism index MMP13, ADAMTS5, and anabolism index COL2 in 4F aging NPCs after doxycycline treatment. (l–n) Immunofluorescence intensity of γH2AX, H3K9me3, and H4K20me3 in 4F aging NPCs after doxycycline treatment. Scale bar, 25 μm. (o–q) Quantification of γH2AX, H3K9me3, and H4K20me3 levels in 4F aging NPCs after doxycycline treatment. Scale bar, 25 μm. (r) ROS levels in 4F aging NPCs after doxycycline treatment. (s) Proliferation of aging NPCs cultured in inducing OSKM lasting 2 or 4 days as determined by EdU incorporation. ****p < 0.0001, according to unpaired two‐sample Student's *t*‐test. Data are presented as mean ± SEM

Hayflick and Moorhead reported replicative senescence (RS) in human dermal fibroblasts (HDFs) (Hayflick & Moorhead, 1961). Notably, NPCs reach the state of RS faster compared with HDFs (Ji et al., 2018). In the present study, the RS model of NPCs (P6) was used to explore whether short‐term induction of OSKM promotes regeneration of aging NPCs. NPCs were isolated from IVD of 4F mice, then cultured to P6 (aging). The short‐term induction of OSKM was performed by doxycycline administration for 2 days (dox2d). The expression of OSKM was confirmed by qPCR and immunofluorescence analysis (Figure 2a,b and Supplementary Figure S2a–d). The results showed that cellular identity was not lost after short‐term induction of OSKM as shown by expression of the NPCs related maker, SOX9. The expression level of SOX9 significantly increased with increase in time of dox induction (Figure 2c). Notably, induction of OSKM did not promote expression of pluripotency marker Nanog for 2 or 4 days (dox4d) (Supplementary Figure S2e). These results indicated that reprogramming did not induce formation of pluripotent stem cells (ipsc). Additionally, aging NPCs showed morphological characteristics for young cells including cell reduction and thickening with decrease in size of the nucleus and nucleoli. Moreover, senescence‐associated β‐galactosidase activity decreased in aging NPCs after short‐term induction of OSKM (Figure 2d). Furthermore, the short‐term induction of OSKM in aging NPCs downregulated expression of age‐related stress response genes in the p53 tumor suppressor pathway, including p16^INK4a^, p21^CIP1^, atf3, and gadd45b (Figure 2e–h). In addition, the induction of OSKM promoted function recovery in aging NPCs, such as boosting anabolism factor col2 expression and downregulating expression of catabolism factor mmp13 and adamts5 (Figure 2i–k). The level of anabolism and catabolism was explored by determination of immunofluorescence of col2 and adamts5 after short‐term induction of OSKM (Supplementary Figure S2m,n). with the findings showed progression of RS process and a significant increase in the positive cells of histone γ‐H2AX indicating nuclear DNA double‐strand breaks associated with aging was observed (Supplementary Figure S2f, Figure 2i). The expression of H3K9me3 and H4K20me3, which are involved in maintenance of heterochromatin showed aging‐related changes (H3K9me3 level showed significant decrease, whereas H4K20me3 showed significant increase in expression level (Supplementary Figure S2g,h,j,k)). The short‐term induction of OSKM in aging NPCs reduced the level of γ‐H2AX compared with that of the control NPCs (Figure 2l,o). Moreover, it restored the levels of H3K9me3 and H4K20me3 to young state (Figure 2m,n,p,q). Apoptosis is a characteristic feature of aging; therefore, the expression level of bcl2, a specific apoptotic protective protein was determined. The findings from Western blot analysis showed a significant increase in expression levels of bcl2 (Supplementary Figure 2l). Notably, the proliferation of aging NPCs was significantly activated (Supplementary Figure S2p and Figure 2s). ROS are responsible for oxidative damage and are major drivers of physiological aging. Short‐term induction of OSKM significantly inhibited production of ROS (Supplementary Figure S2o and Figure 2r). These results indicate that short‐term induction of aging NPCs with OSKM remodels the aging process and promotes activation of a younger state in NPCs.

### 2.3 Maintenance of rejuvenated aging‐associated phenotypes after cyclic induction of OSKM

**FIGURE 3:**
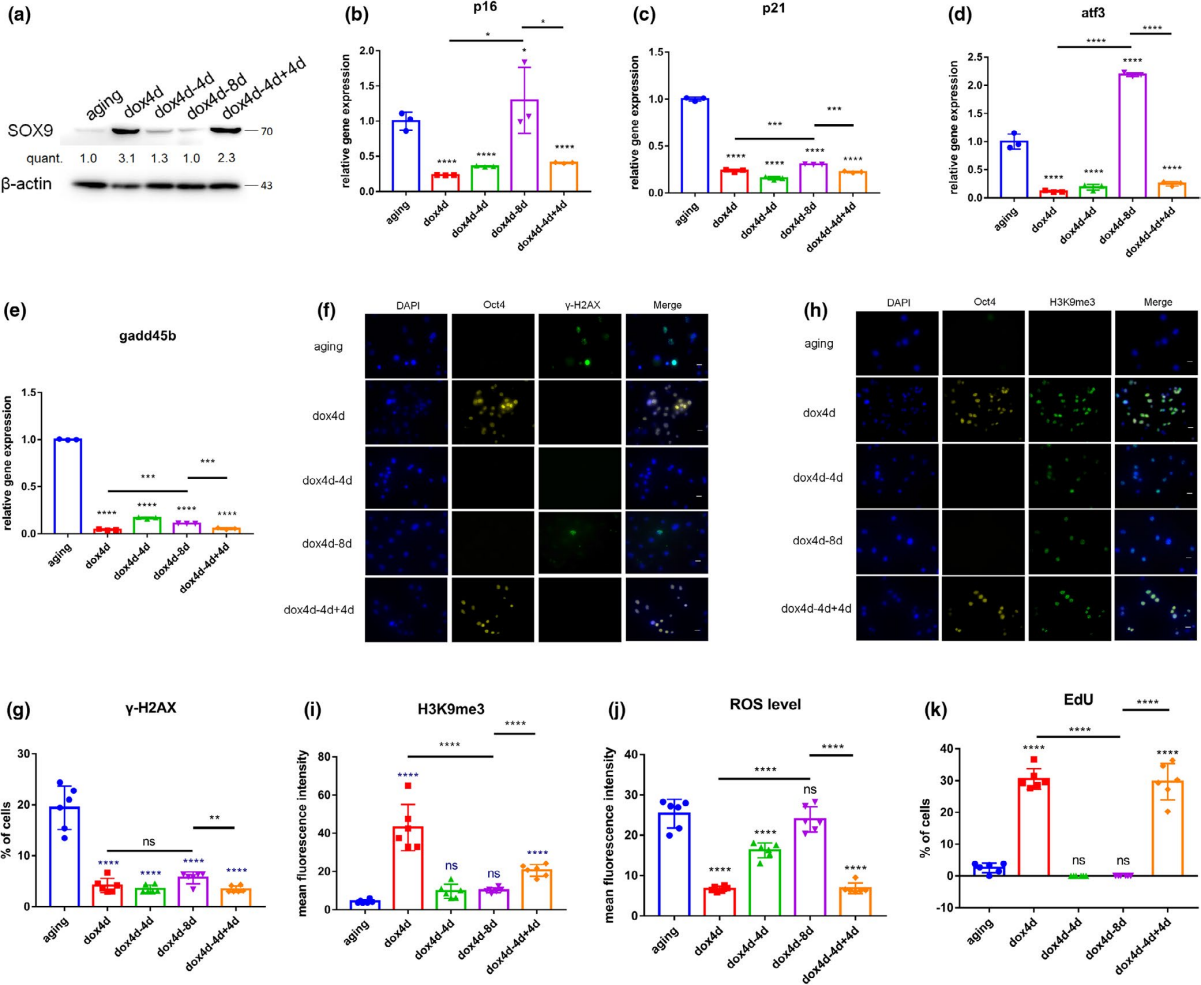
Maintenance of the rejuvenated aging‐associated phenotypes after induction of OSKM. (a) Cultured primary 4F aging NPCs were treated with doxycycline (1 μg/ml) for 4 days (dox4d); 4 days and stopped for 4 days (dox4d‐4d); 4 days and stopped for 8 days (dox4d‐8d); 4 days and stopped for 4 days and then treated for 4 days (dox4d‐4d+4d). Levels of sox9 were determined by Western blotting. (b–e) qPCR analysis of stress response genes in the p53 pathway in 4F aging NPCs after doxycycline treatment. (f,h) Immunofluorescence and quantification of γH2AX expression in 4F aging NPCs after doxycycline treatment. Scale bar, 25 μm. (g,i) Immunofluorescence and quantification of γH2AX and H3K9me3 expression in 4F aging NPCs after doxycycline treatment. Scale bar, 25 μm. (j) Levels of ROS in 4F aging NPCs after doxycycline treatment. (k) Proliferation of aging NPCs cultured in inducing OSKM lasting 2 or 4 days as determined by EdU incorporation. *p < 0.05, **p < 0.01, ***p < 0.001, ****p < 0.0001, according to one‐way ANOVA with Bonferroni correction. Data are presented as mean ± SEM

Doxycycline treatment was stopped at the fourth day for a duration of 4 days (dox4d‐4d) to evaluate the short‐term effect or for a duration of 8 days (dox4d‐8d) for a long‐term effect after induction of OSKM in aging NPCs to explore if the effect was reproducible. In addition, induction was performed for extra 4 days (dox4d‐4d+4d) for a short‐term cyclic induction stage again. The findings showed that cellular identity was not lost after periodic induction of OSKM as shown by the expression of SOX9. Expression of SOX9 increased and decreased after induction and withdrawal of dox induction (Figure 3a). Notably, induction of OSKM did not upregulate expression of pluripotency marker Nanog at any time point (Supplementary Figure S3a). The expression of various age‐related stress response genes was modulated by induction of OSKM. For instance, the expression of p21^CIP1^ and gadd45b was downregulated at dox4d‐4d and dox4d‐8d, whereas the expression of p16^INK4a^ and atf3 was slightly upregulated at dox4d‐8d. Moreover, the expression of p16^INK4a^ and atf3 was downregulated in the dox4d‐4d+4d group (Figure 3b–e). Increase in expression of catabolism factor mmp13 and adamts5 was restored to the aging level in the dox4d‐8d group. Expression level of the anabolism factor col2 was maximum at dox4d‐4d and was restored to the aging level at dox4d‐8d. Notably, dox4d‐4d+4d reversed the effect of short‐term induction of OSKM (Supplementary Figure S3b–d). The analysis of the level of DNA damage (γ‐H2AX) (Figure 3f,g), H3K9me3 (Figure 3h,i), and H4K20me3 (Supplementary Figure S3e,f) at dox4d‐4d and dox4d‐8d revealed a gradual restoration of rejuvenated age‐associated phenotypes. However, dox4d‐4d+4d reversed the aging‐associated phenotypes. Senescence‐associated β‐galactosidase activity was significantly high at dox4d‐4d and dox4d‐8d compared with dox4d. However, dox4d‐4d+4d decreased the level of β‐galactosidase activity (Supplementary Figure S3g). ROS levels showed a similar trend to that of β‐galactosidase activity (Supplementary Figure S3h and Figure 3j). The proliferation of aging NPCs was inhibited when induction was stopped. However, increase in proliferation was observed at dox4d‐4d+4d (Supplementary Figure S3i and Figure 3k). These results demonstrate that maintenance of the ameliorated aging‐associated phenotypes can be achieved when induction is stopped for short periods (like 4 days). However, the effect is lost when induction treatment is terminated for a long period (like 8 days). Notably, short‐term cyclic induction again restores rejuvenation of short‐term induction of OSKM.

### 2.4 Partial reprogramming promotes redistribution of cytoskeleton organization

**FIGURE 4:**
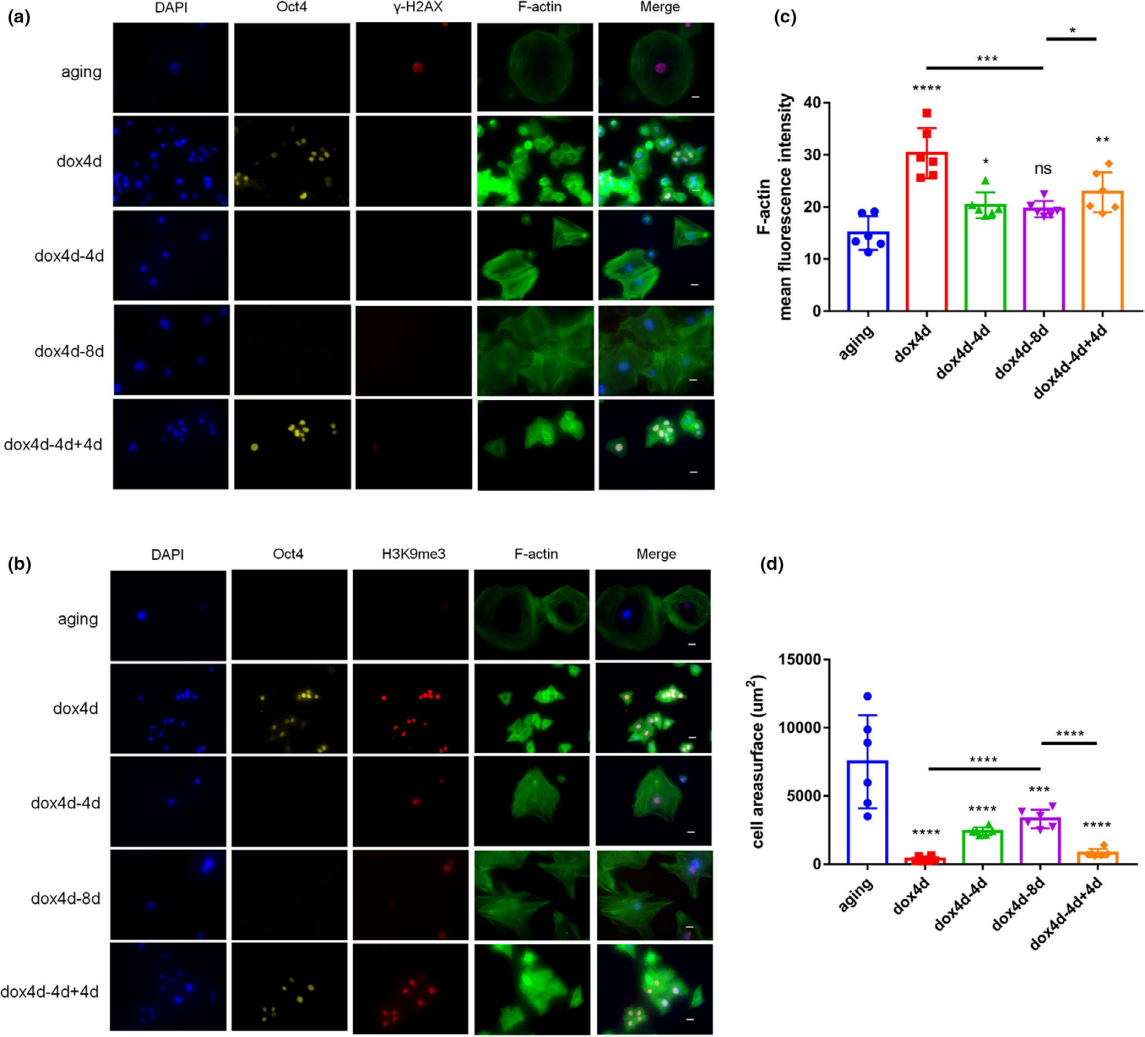
Partial reprogramming promotes redistribution of cytoskeleton organization. (a) Immunofluorescence intensity of γ‐H2AX and F‐actin in 4F aging NPCs during periodicity induction. Scale bar, 25 μm. (b) Immunofluorescence of H3K9me3 and F‐actin in periodic induction during periodic induction. Scale bar, 25 μm. (c) Quantification of F‐actin in 4F aging NPCs during periodic induction. Scale bar, 25 μm. (d) Quantification of cell area surface in 4F aging NPCs during periodic induction. Scale bar, 25 μm. *p < 0.05, **p < 0.01, ***p < 0.001, ****p < 0.0001, according to one‐way ANOVA with Bonferroni correction

The previous findings showed younger morphological change in aging NPCs modulated by short‐term induction of OSKM (Figure 2d). Further analysis was performed to explore whether cytoskeleton was redistributed after the short‐term induction of OSKM. Fluorescent staining of F‐actin and γ‐H2AX revealed higher organized network of fibers in short‐term induction aging NPCs consistent with low level of nuclear DNA double‐strand breaks. Notably, disorganization of F‐actin fibers was observed in aging NPCs which accumulated in the sub‐membrane region (Supplementary Figure S4a). In addition, highly organized cytoskeleton consistent with the significant increase in level of H3K9me3 was observed through immunofluorescence of F‐actin and H3K9me3 (Supplementary Figure S4b and Figure 4c). The short‐term induction of OSKM resulted in lesser cell surface (Supplementary Figure S4d). Short‐term induction of OSKM promoted cytoskeleton formation in aging NPCs. Further analysis on the organization of cytoskeleton at dox4d‐4d and dox4d‐8d was conducted to explore whether the change in cytoskeleton was long‐term. Fluorescent staining of F‐actin and γ‐H2AX (Figure 4a) or H3K9me3 (Figure 4b) showed gradual disorganization of F‐actin fibers. Although the findings showed ordered cytoskeleton at dox4d‐4d, whereas the cells showed significantly disorganized F‐actin fibers at dox4d‐8d. Notably, induction in the dox4d‐4d+4d group restored distribution of fibers. The analysis of mean influorescence intensity of F‐actin showed similar results (Figure 4c). Expression of CDC42 and p70 S6 kinase, which are the regulators of cytoskeleton (Ip et al., 2011; Nobes & Hall, 1995), was significantly upregulated at dox4d and at dox4d‐4d. Notably, the expression was restored to the aging state at dox4d‐8d, and was activated at dox4d‐4d+4d (Supplementary Figure S4e, Figure 4f). The cell surface showed a similar trend in F‐actin fibers after induction with dox (Figure 4d). These findings imply that induction of OSKM promoted redistribution of cytoskeleton organization.

### 2.5 Enhancement of energy metabolism promotes redistribution of cytoskeleton organization in aging NPCs induced by OSKM

**FIGURE 5:**
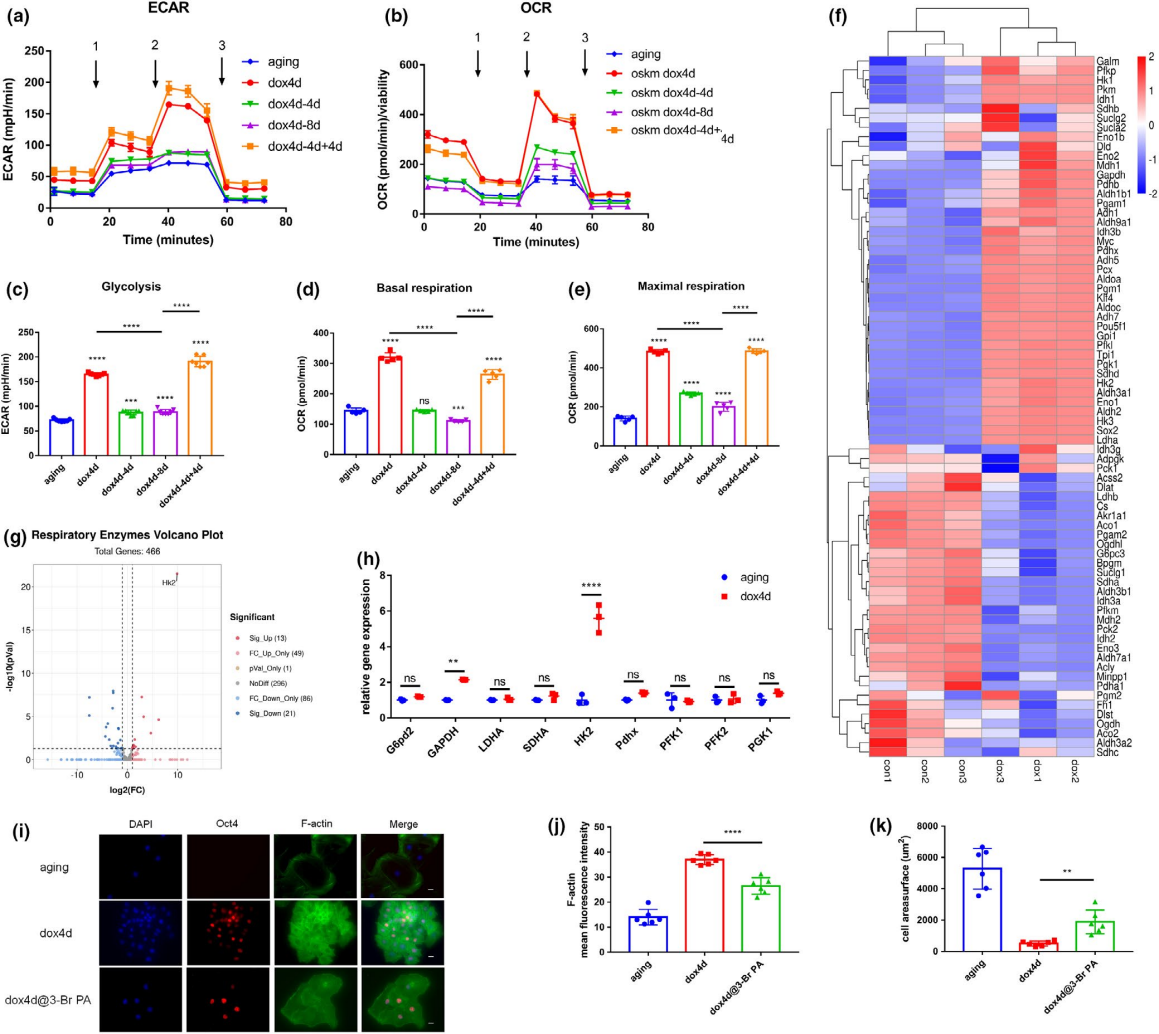
Enhancement of energy metabolism boosts redistribution of cytoskeleton organization in aging NPCs induced by OSKM. (a,b) ECAR determined by glycolysis stress test (Injection 1: Glucose, Injection 2: Oligomycin, Injection 3: 2‐DG), OCR level as determined by MitoStress test (Injection 1: Oligomycin, Injection 2: FCCP, Injection 3: Antimycin A/Rotenone) using Seahorse Instrument. Measurements were performed every 5 min per timepoint for each condition. Data shown in c, d represent mean ± SD for 6 wells, representative of one of the three independent experiments. (c) Quantification of glycolysis from one timepoint in Glycolysis Stress Test (mean ± SD for *n* = 6 wells). (d,e) Quantification of basal respiration and maximal respiration for each timepoint using MitoStress test (mean ± SD for *n* = 6 wells). (f,g) RNA sequencing analysis of 4F aging NPCs treated with doxycycline (1 μg/ml) for 4 days compared with untreated 4F aging NPCs. Heatmap and volcano plots showing expression levels of respiratory enzymes associated genes. (h) qPCR analysis of respiratory enzymes associated genes in glycolysis and tricarboxylic acid cycle (TAC) in 4F aging NPCs. (i,j) Immunofluorescence and quantification of F‐actin in 4F aging NPCs subjected to short‐term expression of OSKM in the presence of 3‐Br PA (HK2 specific inhibitor). Scale bar, 25 μm. (k) Quantification of cell area surface in 4F aging NPCs subjected to short‐term expression of OSKM in the presence of 3‐Br PA (HK2 specific inhibitor). Scale bar, 25 μm. **p < 0.01, ****p < 0.0001 according to one‐way ANOVA with Bonferroni correction

Further, the cause of redistribution of cytoskeleton induced by OSKM in aging NPCs was evaluated. Aging NPCs were induced for 4 days, and RNA‐seq analysis was performed to explore the underlying mechanism of OSKM induced effects. Analysis of genes identified through RNA‐seq analysis revealed a significant difference in several metabolic pathways in the KEGG database. Moreover, gene set analysis significant enrichment of energy metabolic pathways (Supplementary Figure S5). The findings showed significant enrichment of glucose metabolism; therefore, further analysis was conducted to explore if the glucose metabolic shift was mediated by induction of OSKM. Glycolysis was promoted by short‐term induction and reversed to aging level at dox4d‐4d and dox4d‐8d. However, dox4d‐4d+4d activated glycolysis (Figure 5a,c). Notably, OxPhos was activated and showed a significant increase, which is contrary to classical reprogramming. The results showed that dox4d promoted OxPhos process in aging NPCs, and the activation was abrogated at dox4d‐4d and dox4d‐8d, mainly on the level of basic respiration. Maximal respiration gradually decreased at dox4d‐4d and dox4d‐8d. However, dox4d‐4d+4d activated OxPhos (Figure 5b,d,e). Further analysis was conducted to determine the mechanism underlying the enriched energy metabolism. RNA‐seq genes associated with glycolysis and tricarboxylic acid cycle (TAC) were selected for analysis. The findings showed that several respiratory enzymes involved in glycolysis and TAC were significantly upregulated (Figure 5f,g). Analysis showed that hexokinase 2 (HK2) played a crucial role in activating energy metabolism in aging NPCs under the induction of OSKM. The expression of HK2 was significantly upregulated compared with respiratory enzymes (Figure 5h). 3‐Bromopyruvic acid (3‐Br PA), a specific inhibitor of HK2 was used to verify the function of HK2 in redistribution of cytoskeleton (Huang et al., 2020). Immunofluorescence of oct3/4 and F‐actin was performed to determine the level of organization of the cytoskeleton. The results showed more disorganized F‐actin fibers after treatment of NPC cells with 3‐Br PA under induction of dox (Figure 5i). Moreover, a significant decrease in number of fibers was observed (Figure 5j). The cell surface was enlarged after addition of 3‐Br PA (Figure 5k). These results indicate that the activation of HK2 promoted energy metabolism resulting in redistribution of cytoskeleton in aging NPCs which were induced by OSKM.

### 2.6 Enhancement of energy metabolism promotes rejuvenated phenotypes in aging NPCs induced by OSKM

**FIGURE 6:**
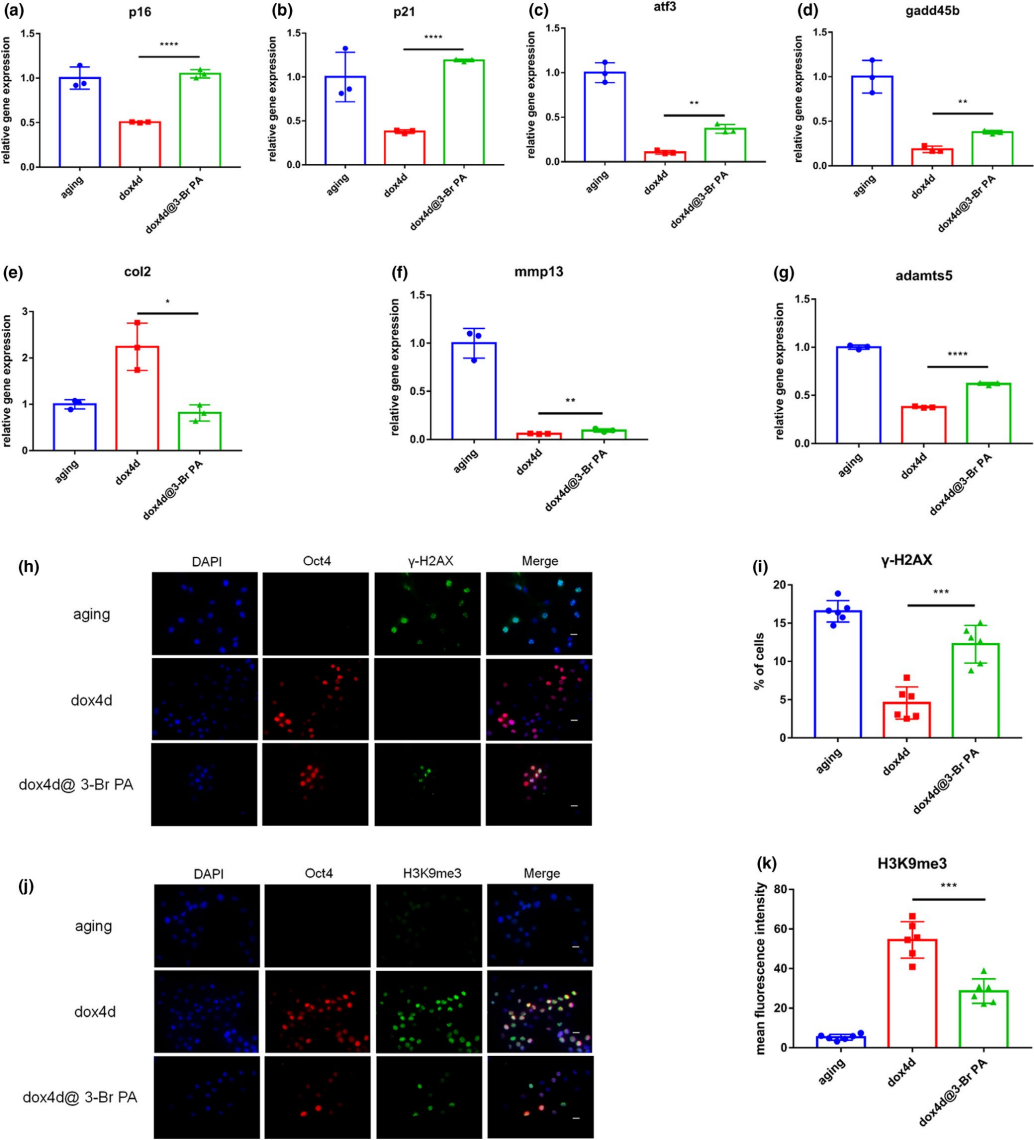
Enhancement of energy metabolism promotes rejuvenated phenotypes in aging NPCs induced by OSKM. (a–d) Expression levels of stress response genes in the p53 pathway in 4F aging NPCs subjected to short‐term induction of OSKM in the presence of 3‐Br PA as determined by qPCR analysis. (e–g) Expression levels of senescence‐associated catabolism index MMP13, ADAMTS5, and anabolism index COL2 in 4F aging NPCs subjected to short‐term expression of OSKM in the presence of 3‐Br PA as determined by qPCR analysis. (h,i) Immunofluorescence and quantification of γH2AX level in 4F aging NPCs subjected to short‐term expression of OSKM in the presence of 3‐Br PA. Scale bar, 25 μm. (j,k) Immunofluorescence and quantification of H3K9me3 level in 4F aging NPCs subjected to short‐term expression of OSKM in the presence of 3‐Br PA. Scale bar, 25 μm. *p < 0.05, **p < 0.01, ***p < 0.001, ****p < 0.0001 according to unpaired two‐sample Student's *t*‐test

Further analysis was conducted to explore whether rejuvenated phenotypes would be obtained by inhibiting activation of HK2. Aging NPCs showed aging morphological changes such as cell enlargement and flattening after addition of 3‐Br PA (Supplementary Figure S6a). Senescence‐associated β‐galactosidase activity significantly increased owing to the inhibitory effect of 3‐Br PA (Supplementary Figure S6a). In addition, the expression levels of age‐related stress response genes, including p16^INK4a^, p21^CIP1^, atf3, and gadd45b, significantly increased after treatment of cells with 3‐Br PA (Figure 6a–d). Furthermore, anabolism and catabolism were reversed, induction was inhibited as shown by the expression levels of col2, mmp13, and adamts5 (Figure 6e–g). Addition of 3‐Br PA to aging NPCs upregulated expression of γ‐H2AX (Figure 6h,i), and restored H3K9me3 to aging level. However, the level of H4K20me3 did not show significant change after addition of 3‐Br PA (Supplementary Figure S6b,c and Figure 6j,k). These results indicate that HK2 plays an important role in promoting rejuvenated phenotypes in aging NPCs induced by OSKM.

## 3 DISCUSSION

Partial reprogramming is a potential strategy to delay senescent process (Ocampo et al., 2016). The findings of the present study showed that partial reprogramming significantly relieved IDD in a needle puncture model and age‐related model. In the current study, 32‐G needle was used to induce changes in neutral zone mechanics of IVD without histology changes, ultimately causing a gradual degeneration process. Notably, 4F mice under dox therapy showed less disc damage, which provides a basis for the development of novel therapies for IDD treatment.

NPCs are the key functional cells that maintain homeostasis of the vertebral disc (Bian et al., 2021). Therefore, analysis was performed to explore the changes in aging NPCs to further understand partial reprogramming in vertebral disc. Physiological aging has similar features as RS, including high levels of DNA damage, changes in chromatin conformation, increased ROS production, decreased proliferation, activated apoptosis and initiation of senescence‐associated secretory phenotype (SASP) (Boulestreau et al., 2021; Guarente, 2008; Haigis & Sinclair, 2010; Kennedy & Lamming, 2016; Soultoukis & Partridge, 2016; Steffen & Dillin, 2016). The phenotypes associated with aging achieve recovery at different degree, through short‐term expression of Yamanaka factors. Moreover, the rejuvenation was achieved through short‐term cyclic induction, indicating that partial reprogramming can rejuvenate aging NPCs. Notably, the identity of aging NPCs was not changed at any time point similar to the findings on partial reprogramming in vitro. SOX9 is a marker for determining the fate and differentiation of chondrocyte lineage (Lefebvre & Dvir‐Ginzberg, 2017). A previous study reported a positive relationship between SOX9 expression, cellularity, and extracellular matrix in disc, implying that SOX9 plays a crucial role in the function of NPCs (Tsingas et al., 2020). SOX9 level showed parallel increase and decrease with the use and withdrawal of OSKM in the present study. This indicates that OSKM significantly upregulates expression of SOX9. Therefore, short‐term induction of OSKM can restore the function of aging NPCs by upregulating SOX9 expression. A significant decrease in levels of catabolism factors such as mmp13 and adamts5 was reported previously (Sarkar et al., 2020). Anabolism factors such as collagen II, which directly reflects the function of NPCs had higher expression at dox2d time point than that at dox4d. Moreover, the higher expression of collagen II was observed short‐term after induction (dox4d‐4d) than the level at dox4d indicating that lasting induction affects expression of collagen II. Induction of OSKM enhanced aging NPCs proliferation. However, long‐term induction was unfavorable for function recovery. Induction‐activated proliferation may impede collagen II synthesis; thus, short‐term cyclic induction should be used to balance proliferation and function recovery in aging NPCs.

Cytoskeleton plays a key role in maintaining cell shape and elasticity as well as cell division and proliferation (Ikeda et al., 2003). Short‐term cyclic induction of Yamanaka factors promotes reorganization of disordered cytoskeleton, and restores cell proliferation (Jeganathan et al., 2016). A well‐organized cytoskeleton in NPCs ensures that discs endure compression stress and prevent IVDD progression (Ke et al., 2021). Partial reprogramming is thus a potential novel method for reorganizing disordered cytoskeleton, and provides new insights into development of effective therapy for inhibiting IVD degeneration. Furthermore, energy metabolism pathway showed significant changes during partial reprogramming in aging NPCs, compared with classical reprogramming (Zhang et al., 2012). Glycolysis and OxPhos pathways were significantly enriched. Cyclic induction results demonstrated that OSKM acted like a “energy switch” to initiate energy metabolism. These changes can be attributed to the aging NPCs state because glycolysis and OxPhos levels are significantly low in aging cells (Azzu & Valencak, 2017). Aging NPCs have no enough energy to achieve rejuvenation unlike young cells in classical reprogramming. Partial reprogramming provides enough energy for the proliferation of aging NPCs. Short‐term induction of OSKM can be used to improve energy metabolism in aging NPCs and rescue its state. RNA sequence analysis between aging NPCs and induced aging NPCs showed that HK2 expression was significantly high in partial reprogramming process, implying that HK2 plays a key role in initiating energy metabolism during partial reprogramming. Therefore, HK2 is a potential target for activating energy metabolism in aging NPCs and for treatment of IDD.

In summary, the findings of the present study show that partial reprogramming through short‐term cyclic induced OSKM in vivo abrogates progression of IDD. Mechanistically, partial reprogramming in vitro promotes energy metabolism as a “energy switch” by upregulating expression of hexokinase 2 (HK2) ultimately promoting redistribution of cytoskeleton and rejuvenating aging state in aging NPCs. Further studies should explore the relationship between proliferation and function recovery, and other effect factors in partial reprogramming to achieve rejuvenation of aging cells and provide a basis for senescence treatment.

## 4 EXPERIMENTAL PROCEDURES

Experimental procedures are described in the Supplementary S2.

## CONFLICT OF INTEREST

The authors declare no competing interests.

## AUTHOR CONTRIBUTIONS

F.C. and C.G.W. conceived the project, designed the experiments, and prepared the manuscript. Y.F.J, J.W.S, K.S.S., and X.P.H. performed the experiments and analyzed the data. S.K.W. and Y.A.Z. performed RNA‐seq analysis. B.Y., K.S.X., and X.P.Z. performed image analysis. L.L.W. provided 4 factor mice, C.Z.L., Q.X.C., and F.C.L. provided key reagents and technical assistance.

## Supporting information

Supplementary Material1

Supplementary Material2

## Data Availability

The data that support the findings of this study are available from the corresponding author upon reasonable request.

## References

[acel13577-bib-0001] Abad, M. , Mosteiro, L. , Pantoja, C. , Cañamero, M. , Rayon, T. , Ors, I. , Graña, O. , Megías, D. , Domínguez, O. , Martínez, D. , Manzanares, M. , Ortega, S. , & Serrano, M. (2013). Reprogramming in vivo produces teratomas and iPS cells with totipotency features. Nature, 502(7471), 340–345. 10.1038/nature12586 24025773

[acel13577-bib-0002] Azzu, V. , & Valencak, T. G. (2017). Energy metabolism and ageing in the mouse: A mini‐review. Gerontology, 63(4), 327–336. 10.1159/000454924 28118636

[acel13577-bib-0003] Bian, J. , Cai, F. , Chen, H. , Tang, Z. , Xi, K. , Tang, J. , Wu, L. , Xu, Y. , Deng, L. , Gu, Y. , Cui, W. , & Chen, L. (2021). Modulation of local overactive inflammation via injectable hydrogel microspheres. Nano Letters, 21(6), 2690–2698. 10.1021/acs.nanolett.0c04713 33543616

[acel13577-bib-0004] Boulestreau, J. , Maumus, M. , Jorgensen, C. , & Noël, D. (2021). Extracellular vesicles from mesenchymal stromal cells: Therapeutic perspectives for targeting senescence in osteoarthritis. Advanced Drug Delivery Reviews, 175, 113836. 10.1016/j.addr.2021.113836 34166759

[acel13577-bib-0005] Global, regional, and national mortality among young people aged 10–24 years, 1950–2019: A systematic analysis for the Global Burden of Disease Study. The Lancet, 398(10311), 1593–1618. 10.1016/s0140-6736(21)01546-4 PMC8576274 34755628

[acel13577-bib-0006] Guarente, L. (2008). Mitochondria–a nexus for aging, calorie restriction, and sirtuins? Cell, 132(2), 171–176. 10.1016/j.cell.2008.01.007 18243090 PMC2680180

[acel13577-bib-0007] Haigis, M. C. , & Sinclair, D. A. (2010). Mammalian sirtuins: Biological insights and disease relevance. Annual Review of Pathology: Mechanisms of Disease, 5, 253–295. 10.1146/annurev.pathol.4.110807.092250 PMC2866163 20078221

[acel13577-bib-0008] Hayflick, L. , & Moorhead, P. S. (1961). The serial cultivation of human diploid cell strains. Experimental Cell Research, 25, 585–621. 10.1016/0014-4827(61)90192-6 13905658

[acel13577-bib-0009] Huang, M. , Xiong, H. , Luo, D. , Xu, B. , & Liu, H. (2020). CSN5 upregulates glycolysis to promote hepatocellular carcinoma metastasis via stabilizing the HK2 protein. Experimental Cell Research, 388(2), 111876. 10.1016/j.yexcr.2020.111876 31991125

[acel13577-bib-0010] Ikeda, S. , Cunningham, L. A. , Boggess, D. , Hawes, N. , Hobson, C. D. , Sundberg, J. P. , Naggert, J. K. , Smith, R. S. , & Nishina, P. M. (2003). Aberrant actin cytoskeleton leads to accelerated proliferation of corneal epithelial cells in mice deficient for destrin (actin depolymerizi.ng factor). Human Molecular Genetics, 12(9), 1029–1037. 10.1093/hmg/ddg112 12700171

[acel13577-bib-0011] Ip, C. K. , Cheung, A. N. , Ngan, H. Y. , & Wong, A. S. (2011). p70 S6 kinase in the control of actin cytoskeleton dynamics and directed migration of ovarian cancer cells. Oncogene, 30(21), 2420–2432. 10.1038/onc.2010.615 21258406

[acel13577-bib-0012] Jeganathan, N. , Predescu, D. , Zhang, J. , Sha, F. , Bardita, C. , Patel, M. , Wood, S. , Borgia, J. A. , Balk, R. A. , & Predescu, S. (2016). Rac1‐mediated cytoskeleton rearrangements induced by intersectin‐1s deficiency promotes lung cancer cell proliferation, migration and metastasis. Molecular Cancer, 15(1), 59. 10.1186/s12943-016-0543-1 27629044 PMC5024437

[acel13577-bib-0013] Ji, M.‐L. , Jiang, H. , Zhang, X.‐J. , Shi, P.‐L. , Li, C. , Wu, H. , Wu, X.‐T. , Wang, Y.‐T. , Wang, C. , & Lu, J. (2018). Preclinical development of a microRNA‐based therapy for intervertebral disc degeneration. Nature Communications, 9(1), 5051. 10.1038/s41467-018-07360-1 PMC6262020 30487517

[acel13577-bib-0014] Ke, W. , Wang, B. , Hua, W. , Song, Y. U. , Lu, S. , Luo, R. , Li, G. , Wang, K. , Liao, Z. , Xiang, Q. , Li, S. , Wu, X. , Zhang, Y. , & Yang, C. (2021). The distinct roles of myosin IIA and IIB under compression stress in nucleus pulposus cells. Cell Proliferation, 54(2), e12987. 10.1111/cpr.12987 33415745 PMC7848961

[acel13577-bib-0015] Kennedy, B. K. , & Lamming, D. W. (2016). The mechanistic target of rapamycin: The grand ConducTOR of metabolism and aging. Cell Metabolism, 23(6), 990–1003. 10.1016/j.cmet.2016.05.009 27304501 PMC4910876

[acel13577-bib-0016] Lefebvre, V. , & Dvir‐Ginzberg, M. (2017). SOX9 and the many facets of its regulation in the chondrocyte lineage. Connective Tissue Research, 58(1), 2–14. 10.1080/03008207.2016.1183667 27128146 PMC5287363

[acel13577-bib-0017] Lu, Y. , Brommer, B. , Tian, X. , Krishnan, A. , Meer, M. , Wang, C. , Vera, D. L. , Zeng, Q. , Yu, D. , Bonkowski, M. S. , Yang, J.‐H. , Zhou, S. , Hoffmann, E. M. , Karg, M. M. , Schultz, M. B. , Kane, A. E. , Davidsohn, N. , Korobkina, E. , Chwalek, K. , … Sinclair, D. A. (2020). Reprogramming to recover youthful epigenetic information and restore vision. Nature, 588(7836), 124–129. 10.1038/s41586-020-2975-4 33268865 PMC7752134

[acel13577-bib-0018] Madhu, V. , Boneski, P. K. , Silagi, E. , Qiu, Y. , Kurland, I. , Guntur, A. R. , Shapiro, I. M. , & Risbud, M. V. (2020). Hypoxic regulation of mitochondrial metabolism and mitophagy in nucleus pulposus cells is dependent on HIF‐1α‐BNIP3 axis. Journal of Bone and Mineral Research, 35(8), 1504–1524. 10.1002/jbmr.4019 32251541 PMC7778522

[acel13577-bib-0020] Murgatroyd, C. , Wu, Y. , Bockmühl, Y. , & Spengler, D. (2010). The Janus face of DNA methylation in aging. Aging (Albany NY), 2(2), 107–110. 10.18632/aging.100124 20354272 PMC2850147

[acel13577-bib-0021] Nobes, C. D. , & Hall, A. (1995). Rho, rac, and cdc42 GTPases regulate the assembly of multimolecular focal complexes associated with actin stress fibers, lamellipodia, and filopodia. Cell, 81(1), 53–62. 10.1016/0092-8674(95)90370-4 7536630

[acel13577-bib-0022] Ocampo, A. , Reddy, P. , Martinez‐Redondo, P. , Platero‐Luengo, A. , Hatanaka, F. , Hishida, T. , Li, M. O. , Lam, D. , Kurita, M. , Beyret, E. , Araoka, T. , Vazquez‐Ferrer, E. , Donoso, D. , Roman, J. L. , Xu, J. , Rodriguez Esteban, C. , Nuñez, G. , Nuñez Delicado, E. , Campistol, J. M. , … Izpisua Belmonte, J. C. (2016). In vivo amelioration of age‐associated hallmarks by partial reprogramming. Cell, 167(7), 1719–1733.e1712. 10.1016/j.cell.2016.11.052 27984723 PMC5679279

[acel13577-bib-0023] Ohnishi, K. , Semi, K. , Yamamoto, T. , Shimizu, M. , Tanaka, A. , Mitsunaga, K. , Okita, K. , Osafune, K. , Arioka, Y. , Maeda, T. , Soejima, H. , Moriwaki, H. , Yamanaka, S. , Woltjen, K. , & Yamada, Y. (2014). Premature termination of reprogramming in vivo leads to cancer development through altered epigenetic regulation. Cell, 156(4), 663–677. 10.1016/j.cell.2014.01.005 24529372

[acel13577-bib-0024] Polo, J. , Anderssen, E. , Walsh, R. , Schwarz, B. , Nefzger, C. , Lim, S. , Borkent, M. , Apostolou, E. , Alaei, S. , Cloutier, J. , Bar‐Nur, O. , Cheloufi, S. , Stadtfeld, M. , Figueroa, M. , Robinton, D. , Natesan, S. , Melnick, A. , Zhu, J. , Ramaswamy, S. , & Hochedlinger, K. (2012). A molecular roadmap of reprogramming somatic cells into iPS cells. Cell, 151(7), 1617–1632. 10.1016/j.cell.2012.11.039 23260147 PMC3608203

[acel13577-bib-0025] Roberts, S. , Evans, H. , Trivedi, J. , & Menage, J. (2006). Histology and pathology of the human intervertebral disc. Journal of Bone and Joint Surgery. American Volume, 88(Suppl 2), 10–14. 10.2106/jbjs.F.00019 16595436

[acel13577-bib-0026] Rodríguez‐Matellán, A. , Alcazar, N. , Hernández, F. , Serrano, M. , & Ávila, J. (2020). In vivo reprogramming ameliorates aging features in dentate gyrus cells and improves memory in mice. Stem Cell Reports, 15(5), 1056–1066. 10.1016/j.stemcr.2020.09.010 33096049 PMC7663782

[acel13577-bib-0027] Sakai, D. , & Grad, S. (2015). Advancing the cellular and molecular therapy for intervertebral disc disease. Advanced Drug Delivery Reviews, 84, 159–171. 10.1016/j.addr.2014.06.009 24993611

[acel13577-bib-0028] Sarkar, T. J. , Quarta, M. , Mukherjee, S. , Colville, A. , Paine, P. , Doan, L. , Tran, C. M. , Chu, C. R. , Horvath, S. , Qi, L. S. , Bhutani, N. , Rando, T. A. , & Sebastiano, V. (2020). Transient non‐integrative expression of nuclear reprogramming factors promotes multifaceted amelioration of aging in human cells. Nature Communications, 11(1), 1545. 10.1038/s41467-020-15174-3 PMC7093390 32210226

[acel13577-bib-0029] Shibata, H. , Komura, S. , Yamada, Y. , Sankoda, N. , Tanaka, A. , Ukai, T. , Kabata, M. , Sakurai, S. , Kuze, B. , Woltjen, K. , Haga, H. , Ito, Y. , Kawaguchi, Y. , Yamamoto, T. , & Yamada, Y. (2017). Hybrid cellular metabolism coordinated by Zic3 and Esrrb synergistically enhances induction of naive pluripotency. Cell Metabolism, 25(5), 1103–1117.e1106. 10.1016/j.cmet.2017.04.017 28467928

[acel13577-bib-0030] Shibata, H. , Komura, S. , Yamada, Y. , Sankoda, N. , Tanaka, A. , Ukai, T. , Kabata, M. , Sakurai, S. , Kuze, B. , Woltjen, K. , Haga, H. , Ito, Y. , Kawaguchi, Y. , Yamamoto, T. , & Yamada, Y. (2018). In vivo reprogramming drives Kras‐induced cancer development. Nature Communications, 9(1), 2081. 10.1038/s41467-018-04449-5 PMC5970190 29802314

[acel13577-bib-0031] Soultoukis, G. A. , & Partridge, L. (2016). Dietary protein, metabolism, and aging. Annual Review of Biochemistry, 85, 5–34. 10.1146/annurev-biochem-060815-014422 27145842

[acel13577-bib-0032] Steffen, K. K. , & Dillin, A. (2016). A ribosomal perspective on proteostasis and aging. Cell Metabolism, 23(6), 1004–1012. 10.1016/j.cmet.2016.05.013 27304502

[acel13577-bib-0033] Takahashi, K. , Tanabe, K. , Ohnuki, M. , Narita, M. , Ichisaka, T. , Tomoda, K. , & Yamanaka, S. (2007). Induction of pluripotent stem cells from adult human fibroblasts by defined factors. Cell, 131(5), 861–872. 10.1016/j.cell.2007.11.019 18035408

[acel13577-bib-0034] Takahashi, K. , & Yamanaka, S. (2006). Induction of pluripotent stem cells from mouse embryonic and adult fibroblast cultures by defined factors. Cell, 126(4), 663–676. 10.1016/j.cell.2006.07.024 16904174

[acel13577-bib-0035] Tsingas, M. , Ottone, O. K. , Haseeb, A. , Barve, R. A. , Shapiro, I. M. , Lefebvre, V. , & Risbud, M. V. (2020). Sox9 deletion causes severe intervertebral disc degeneration characterized by apoptosis, matrix remodeling, and compartment‐specific transcriptomic changes. Matrix Biology, 94, 110–133. 10.1016/j.matbio.2020.09.003 33027692 PMC7778523

[acel13577-bib-0036] Wang, J. , Tian, Y. E. , Phillips, K. L. E. , Chiverton, N. , Haddock, G. , Bunning, R. A. , Cross, A. K. , Shapiro, I. M. , Le Maitre, C. L. , & Risbud, M. V. (2013). Tumor necrosis factor α‐ and interleukin‐1β‐dependent induction of CCL3 expression by nucleus pulposus cells promotes macrophage migration through CCR1. Arthritis and Rheumatism, 65(3), 832–842. 10.1002/art.37819 23233369 PMC3582738

[acel13577-bib-0037] Zhang, J. , Nuebel, E. , Daley, G. Q. , Koehler, C. M. , & Teitell, M. A. (2012). Metabolic regulation in pluripotent stem cells during reprogramming and self‐renewal. Cell Stem Cell, 11(5), 589–595. 10.1016/j.stem.2012.10.005 23122286 PMC3492890

