# Supplementary material for: Partial reprogramming strategy for intervertebral disc rejuvenation by activating energy switch
Source: Aging Cell. 2022 Mar 9;21(4):e13577. doi: 10.1111/acel.13577 (PMC9009234; doi:10.1111/acel.13577)
Supplement: Supplementary file 1 — Supplementary Material1 [file ACEL-21-e13577-s002.docx]

**Supplementary Information**

**Partial reprogramming strategy for intervertebral disc rejuvenation by activating energy switch**

Feng Cheng^1, 2, 3#^, Chenggui Wang^4#^, Yufei Ji^5^, Biao Yang^1, 2, 3^, Jiawei Shu^1, 2, 3^, Kesi Shi^1, 2, 3^, Lulu Wang^6^, Shaoke Wang^1, 2, 3^, Yuang Zhang^1, 2, 3^, Xianpeng Huang^1, 2, 3^, Xiaopeng Zhou^1, 2, 3^, Kaishun Xia^1, 2, 3^, Chengzhen Liang^1, 2, 3*^, Qixin Chen^1, 2, 3*^, and Fangcai Li^1, 2, 3*^

^1^ Department of Orthopedics Surgery, the Second Affiliated Hospital, School of Medicine, Zhejiang University, Hangzhou 310009, Zhejiang, China

^2^ Orthopedics Research Institute of Zhejiang University, Hangzhou 310009, Zhejiang, China

^3^ Key Laboratory of Motor System Disease Research and Precision Therapy of Zhejiang Province, Hangzhou 310009, Zhejiang, China

^4^ Department of Orthopedics, The Second Affiliated Hospital and Yuying Children’s Hospital of Wenzhou Medical University, Wenzhou, 325000, Zhejiang Province, China

^5^Department of Gastrointestinal Surgery, Xiamen Cancer Center, The First Affiliated Hospital of Xiamen University, Xiamen, China

^6^ Laboratory of Metabolism and Cell Fate, Guangzhou Institutes of Biomedicine and Health, Chinese Academy of Sciences, 510530 Guangzhou, China.

#These authors contributed equally to this work

*Correspondence should be addressed to Chengzhen Liang (liangchengzhen@zju.edu.cn), Qixin Chen (zrcqx@zju.edu.cn), and Fangcai Li (lifangcai@zju.edu.cn)

**
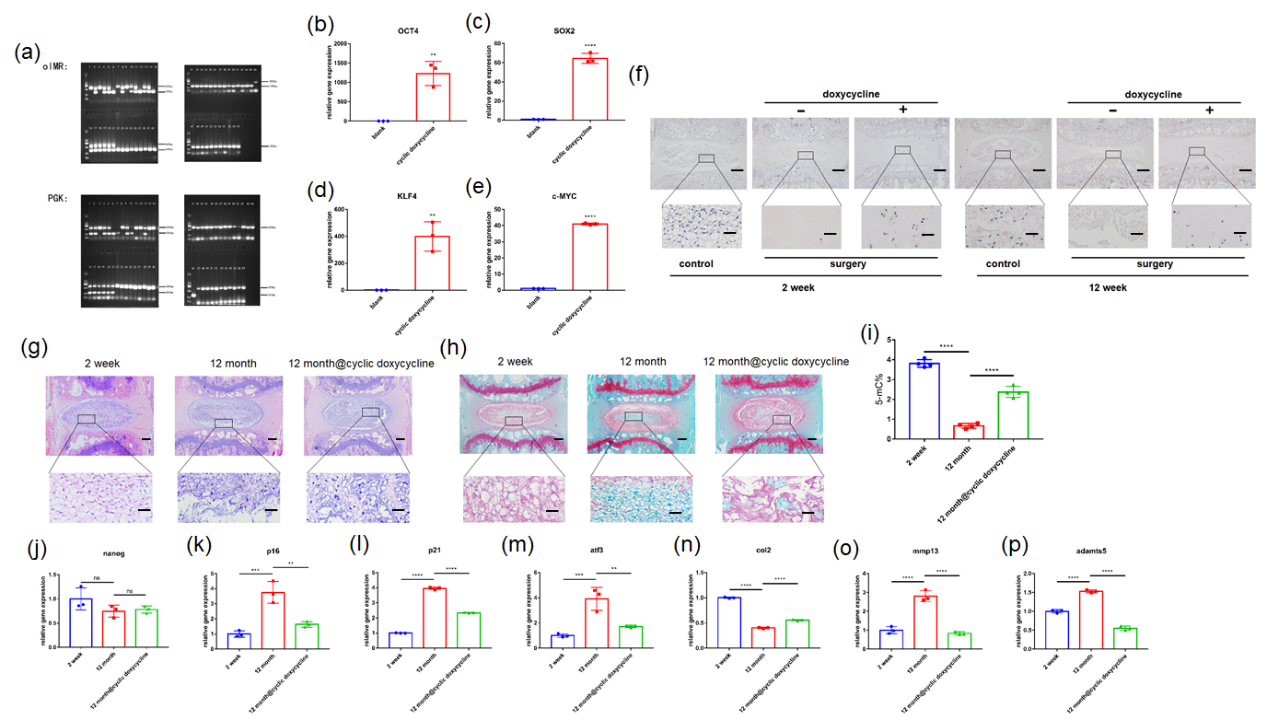
**

**Supplementary Fig. 1 Age-related improvement in 4 factor mice after short-term cyclic reprogramming *in vivo*.** (a) PCR analysis of genotype including olMR and PGK in 4F mice. (b-e) qPCR analysis of Oct4, Sox2, Klf4, and c-Myc in nucleus pulposus of 4F mice. **p< 0.01, ****p< 0.0001, according to unpaired two-sample Student’s t test. (f) Immunohistochemistry of col2 of disc sections in control groups and treatment groups at week 4 and week 14 after surgery and blank groups (water groups, n=5; doxycycline groups, n=5; blank groups, n=5). Scale bar=50μm. (g) HE staining of disc sections in 2 week groups and 12 month groups and 12 month@cyclic doxycycline groups (2 week groups, n=5; 12 month groups, n=5; 12 month@cyclic doxycycline, n=5). Scale bar=50μm. (h) Safranin O staining of disc sections in 2 week groups and 12 month groups and 12 month@cyclic doxycycline groups, Scale bar=50μm. (i) level of 5-mc in 2 week groups and 12 month groups and 12 month@cyclic doxycycline. (j-p) qPCR analysis of nanog, p16, p21, atf3, col2, mmp13 and adamts5 in nucleus pulposus of 2 week groups and 12 month groups and 12 month@cyclic doxycycline groups. **p< 0.01, ***p< 0.001, ****p< 0.0001, according to unpaired two-sample Student’s t test.


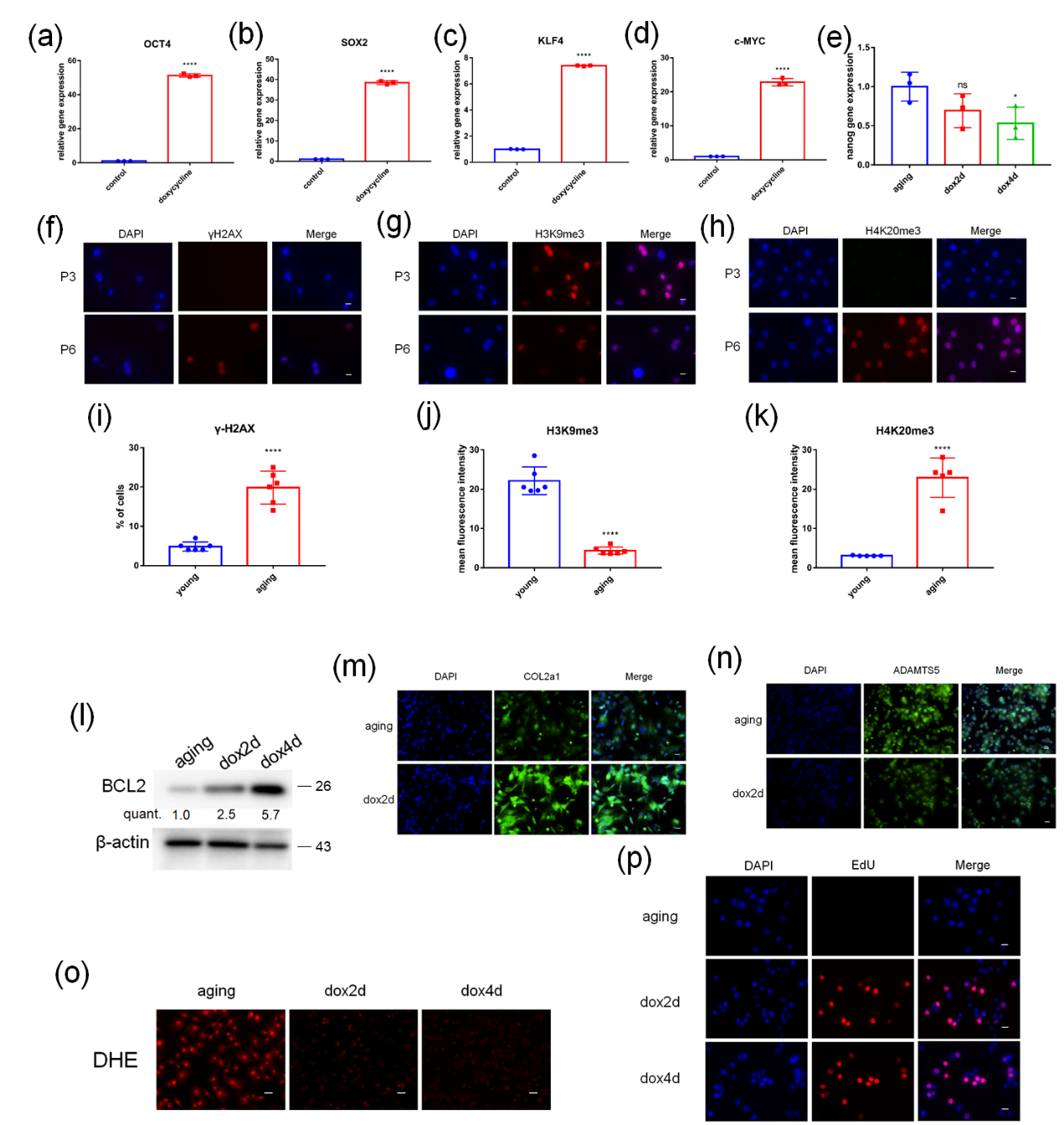


**Supplementary Fig. 2 Partial reprogramming rejuvenates phenotypes associated with aging in degenerative nucleus pulposus cells** (a-d) qPCR analysis of Oct4, Sox2, Klf4, and c-Myc in aging NPCs after doxycycline treatment. ****p< 0.0001, according to unpaired two-sample Student’s t test. (e) qPCR analysis of nanog in aging NPCs after doxycycline treatment, *p< 0.05, according to unpaired two-sample Student’s t test. (f-h) Immunofluorescence of γH2AX, H3K9me3 and H4K20me3 in young or aging NPCs. Scale bar, 25 μm. (i-k) Quantification of γH2AX, H3K9me3 and H4K20me3 in young or aging NPCs. Scale bar, 25 μm. (l) Cultured primary 4F aging NPCs were treated with doxycycline (1 μg/ml) for 2 days (dox2d) and 4 days (dox4d), then the levels of bcl2 were measured by western blotting. (m,n) Immunofluorescence of col2 and adamts5 in aging NPCs. Scale bar, 25 μm. (o) ROS in 4F aging NPCs after doxycycline treatment. Scale bar, 25 μm. (p) Proliferation, as determined by EdU incorporation, of aging NPCs cultured in inducing OSKM lasting 2 or 4 days. Scale bar, 25 μm.


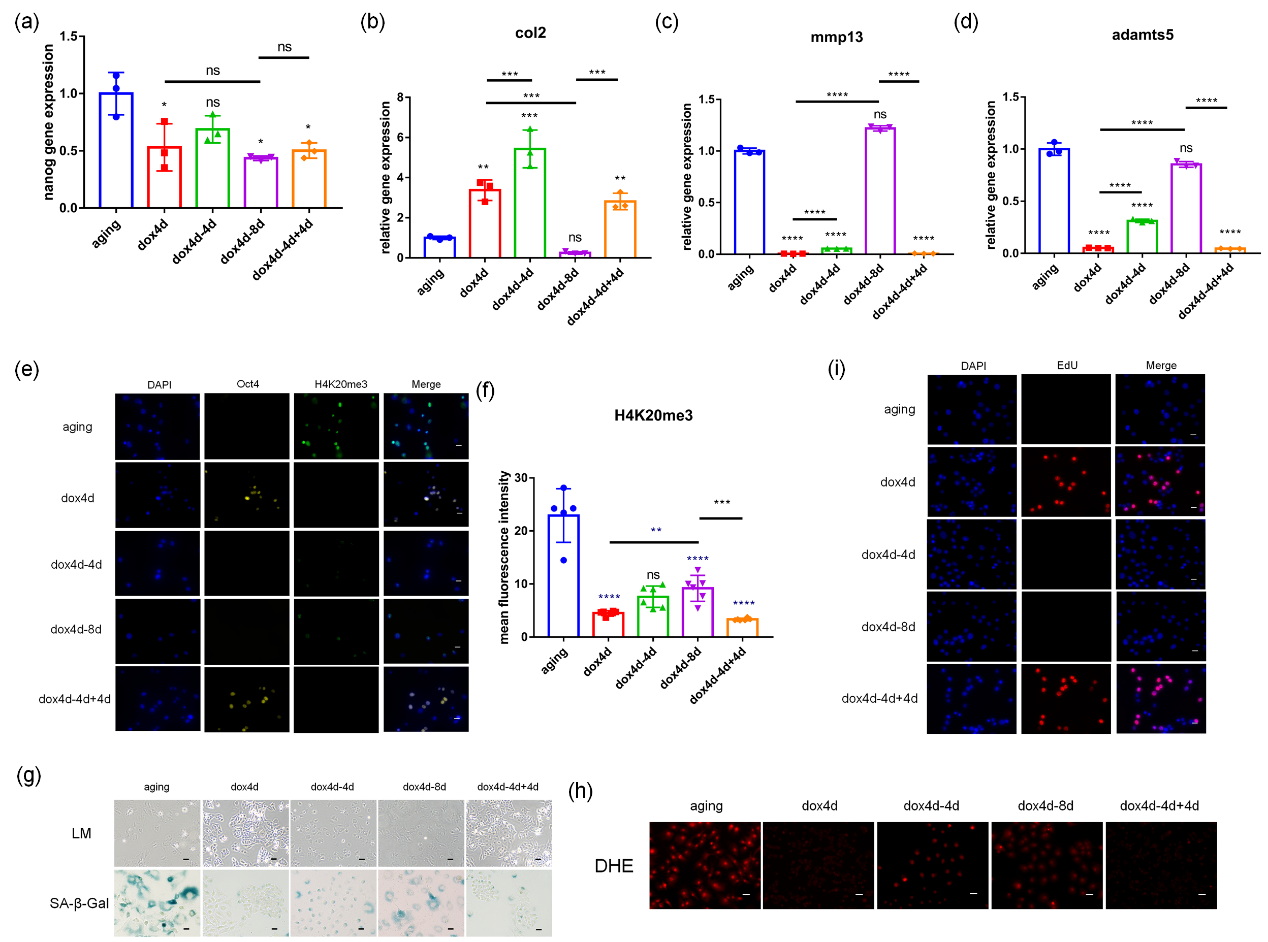


**Supplementary Fig. 3 Maintenance of the rejuvenated aging-associated phenotypes after Induction of OSKM.** (a) qPCR analysis of nanog in aging NPCs after doxycycline treatment, *p< 0.05, according to unpaired two-sample Student’s t test. (b-d) qPCR analysis of senescence-associated catabolism index MMP13, ADAMTS5 and anabolism index COL2 in 4F aging NPCs after doxycycline treatment. **p < 0.01, ***p < 0.001, ****p < 0.0001, according to one-way ANOVA with Bonferroni correction. (e,f) Immunofluorescence and quantification of H4K20me3 in 4F aging NPCs after doxycycline treatment. Scale bar, 25 μm. **p < 0.01, ***p < 0.001, **** p < 0.0001, according to one-way ANOVA with Bonferroni correction. (g) Light microscope (LM) and β-galactosidase activity in aging, dox4d, dox4d-4d, dox4d-8d and dox4d-4d+4d. LM, scale bar, 100 μm; β-galactosidase staining, scale bar, 50 μm. (h) ROS in 4F aging NPCs after doxycycline treatment. Scale bar, 25 μm. i Proliferation, as determined by EdU incorporation, of aging NPCs cultured in dox4d, dox4d-4d, dox4d-8d and dox4d-4d+4d. Scale bar, 25 μm.


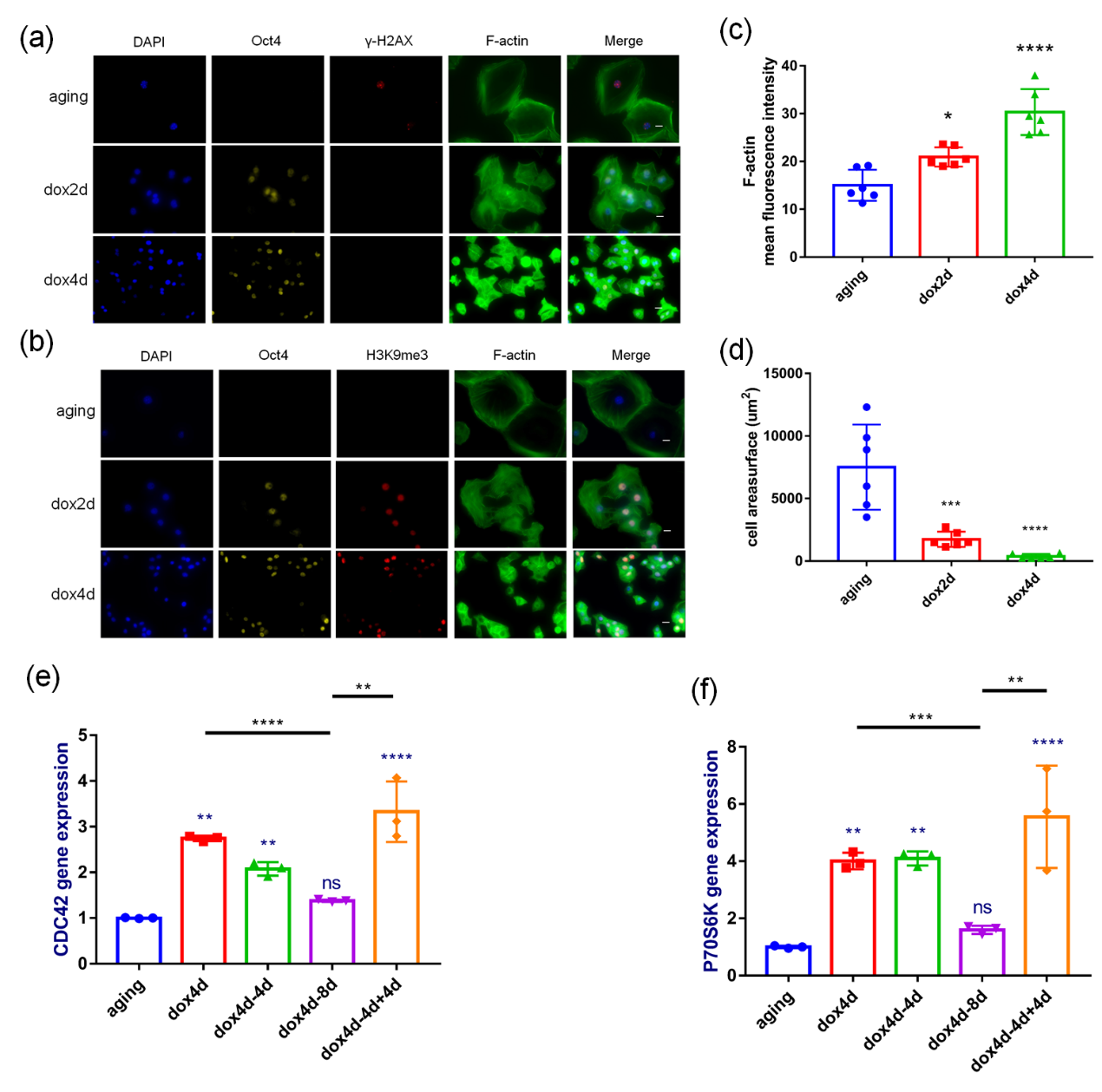


**Supplementary Fig. 4 Partial reprogramming promotes the redistribution of cytoskeleton organization.** (a) Immunofluorescence of γ-H2AX and F-actin in 4F aging NPCs after doxycycline treatment. Scale bar, 25 μm. (b) Immunofluorescence of H3K9me3 and F-actin in periodicity induction after doxycycline treatment. Scale bar, 25 μm. (c) Quantification of F-actin in 4F aging NPCs after doxycycline treatment. Scale bar, 25 μm. * p < 0.05, **** p < 0.0001, according to unpaired two-sample Student’s t test. (d) Quantification of cell area surface in 4F aging NPCs during periodicity induction. Scale bar, 25 μm. *** p < 0.001, **** p < 0.0001, according to unpaired two-sample Student’s t test. (e,f) qPCR analysis of CDC42 and P70S6k in aging NPCs after doxycycline treatment, **p< 0.01, *** p < 0.001, **** p < 0.0001, according to one-way ANOVA with Bonferroni correction.

**
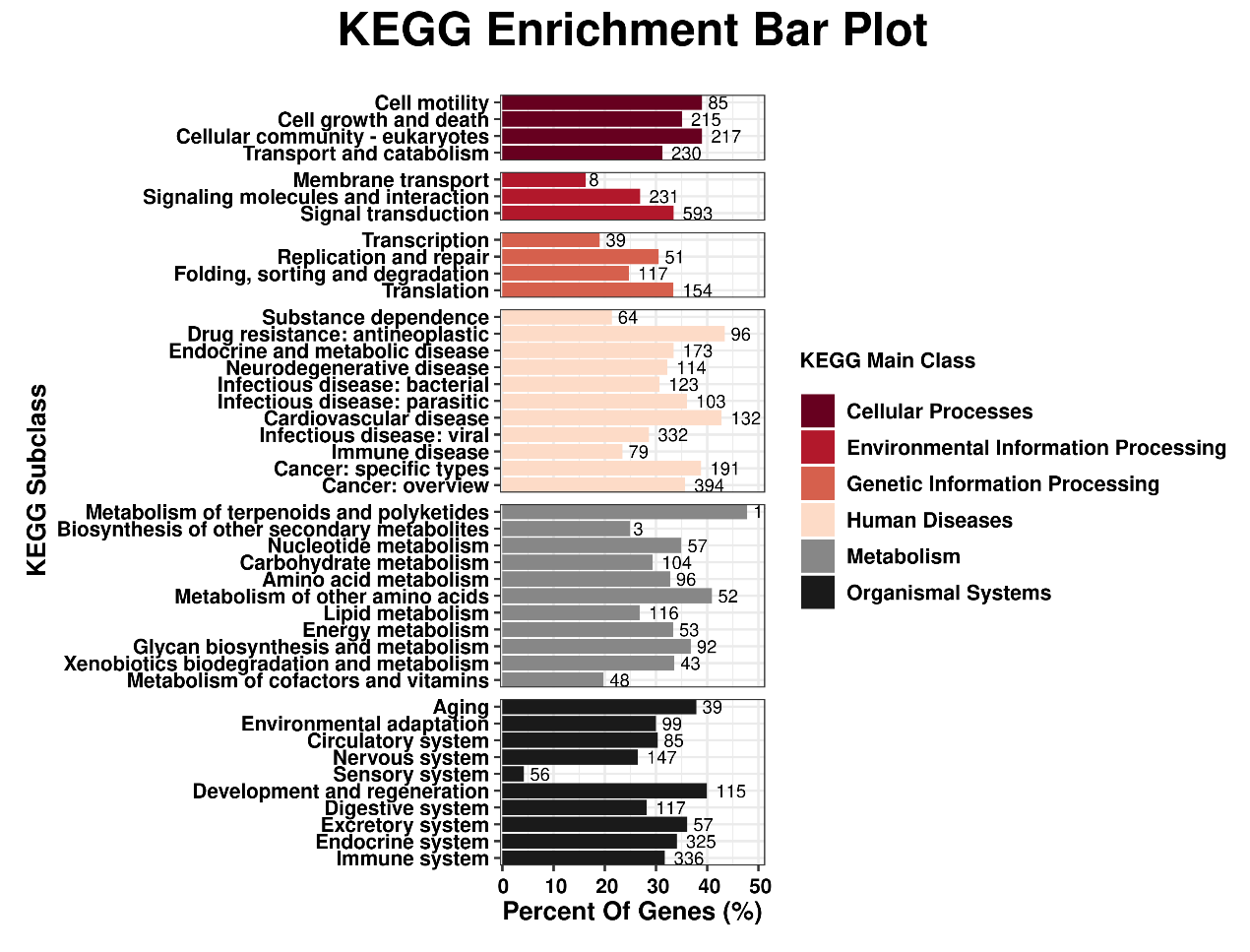
**

**Supplementary Fig. 5 KEGG analysis between induced 4F aging NPCs and untreated 4F aging NPCs.**


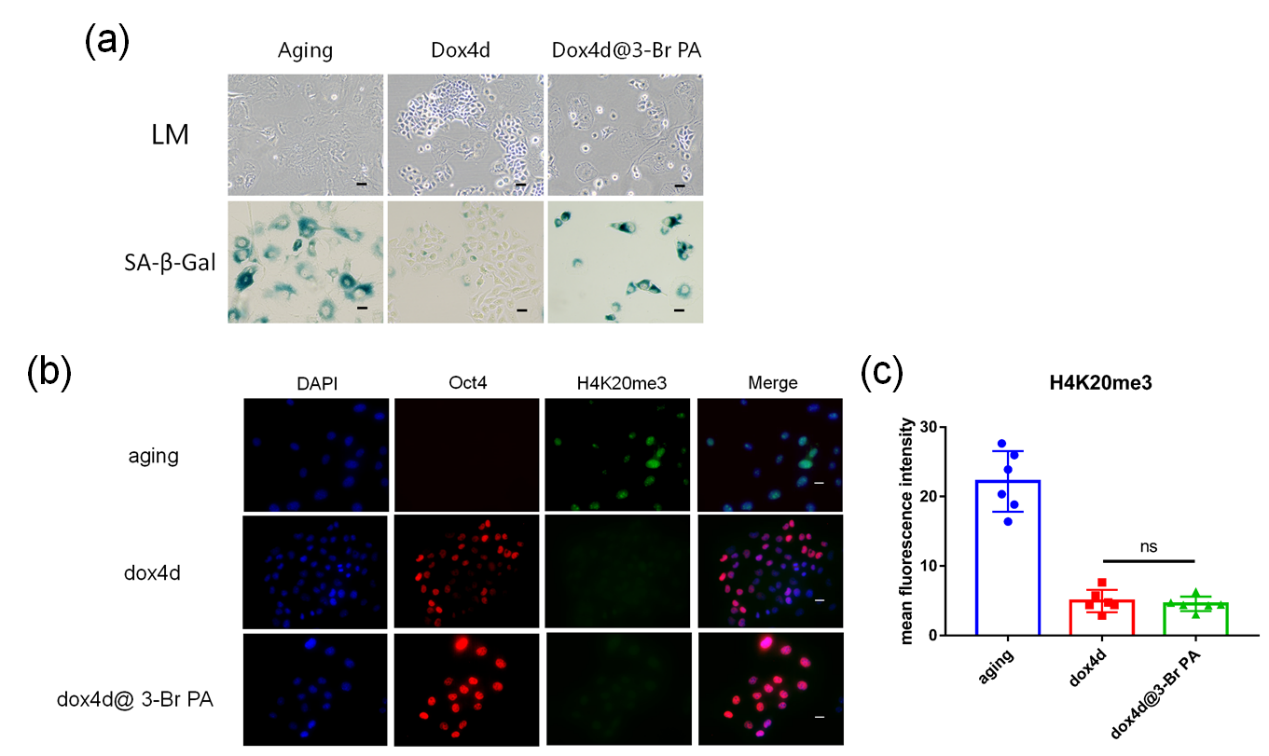


**Supplementary Fig. 6 Enhancement of energy metabolism promotes rejuvenated phenotypes in aging NPCs by induction of OSKM.** (a) Light microscope (LM) and β-galactosidase activity in aging, dox4d and dox4d@3-Br PA. LM, scale bar, 100 μm; β-galactosidase staining, scale bar, 50 μm. (b,c) Immunofluorescence and quantification of H4K20me3 in 4F aging NPCs subjected to short-term expression of OSKM in the presence of 3-Br PA. Scale bar, 25 μm.

**Supplementary Table**

| Gene Name | 5'-Sequence-3' |
| --- | --- |
| olMR8052 | GCGAAGAGTTTGTCCTCAACC |
| olMR8545 | AAAGTCGCTCTGAGTTGTTAT |
| olMR8546 | GGAGCGGGAGAAATGGATATG |
| 4F2A-Col1a1-f | CCAGTTCAATCATCCCAGGTGC |
| 4F2A-Col1a1-r | TCCCTGTTTCTGCTGCTTGA |
| 4F2A-PGK | CCAGAAAGCGAAGGAGCAAAG |
| Oct4-f | GGCTTCAGACTTCGCCTTCT |
| Oct4-r | TGGAAGCTTAGCCAGGTTCG |
| Sox2-f | TTTGTCCGAGACCGAGAAGC |
| Sox2-r | CTCCGGGAAGCGTGTACTTA |
| Klf4-f | GCACACCTGCGAACTCACAC |
| Klf4-r | CCGTCCCAGTCACAGTGGTAA |
| c-Myc-f | ACCACCAGCAGCGACTCTGA |
| c-Myc-r | TGCCTCTTCTCCACAGACACC |
| P16-f | CGTGAACATGTTGTTGAGGC |
| P16-r | GCAGAAGAGCTGCTACGTGA |
| P21-f | CGGTGTCAGAGTCTAGGGGA |
| P21-r | ATCACCAGGATTGGACATGG |
| Atf3-f | CTCTGGCCGTTCTCTGGA |
| Atf3-r | GGTCGCACTGACTTCTGAGG |
| Gadd45b-f | CGGCCAAACTGATGAATGT |
| Gadd45b-r | TCTGCAGAGCGATATCATCC |
| Col2-f | CCAAACCAGCCTGACAACTT |
| Col2-r | TCTAGCATGCTCCACCACTG |
| Mmp13-f | TTTGAGAACACGGGGAAGA |
| Mmp13-r | ACTTTGTTGCCAATTCCAGG |
| Adamts5-f | CCAAATGCACTTCAGCCACGATCA |
| Adamts5-r | AATGTCAAGTTGCACTGCTGGGTG |
| Nanog-f | CAGGTGTTTGAGGGTAGCT |
| Nanog-r | CGGTTCATCATGGTACAGTC |
| Cdc42-f | CCCATCGGAATATGTACCAACTG |
| Cdc42-r | CCAAGAGTGTATGGCTCTCCAC |
| P70S6K-f | GGGGCTATGGAAAGGTTTTTCA |
| P70S6K-r | CGTGTCCTTAGCATTCCTCACT |
| Actin-f | CTAAGGCCAACCGTGAAAAG |
| Actin-r | ACCAGAGGCATACAGGGACA |
| G6pd2-f | CTGAATGAACGCAAAGCTGA |
| G6pd2-r | CAATCTTGTGCAGCAGTGGT |
| Gapdh-f | GGCAAATTCAACGGCACAGT |
| Gapdh-r | GTCTCGCTCCTGGAAGATGG |
| Ldha-f | GGATGAGCTTGCCCTTGTTGA |
| Ldha-r | GACCAGCTTGGAGTTCGCAGTTA |
| Sdha-f | AACACTGGAGGAAGCACACC |
| Sdha-r | AGTAGGAGCGGATAGCAGGA |
| Hk2-f | TGATCGCCTGCTTATTCACGG |
| Hk2-r | AACCGCCTAGAAATCTCCAGA |
| Pdhx-f | GCTTCACTGTAACCAGCCG |
| Pdhx-r | CCCTTGCTCCATCGTAGGAGA |
| Pfk1-f | GAGCGAGAAGGACGACTCC |
| Pfk1-r | GCCTCCGATGACACACAGA |
| Pfk2-f | TGTGGTCCGAGTTGGTATCTT |
| Pfk2-r | GCACTTCCAATCACTGTGCC |
| Pgk-f | ATGTCGCTTTCCAACAAGCTG |
| Pgk-r | GCTCCATTGTCCAAGCAGAAT |
