# Supplementary material for: Partial reprogramming strategy for intervertebral disc rejuvenation by activating energy switch
Source: Aging Cell. 2022 Mar 9;21(4):e13577. doi: 10.1111/acel.13577 (PMC9009234; doi:10.1111/acel.13577)
Supplement: Supplementary file 2 — Supplementary Material2 [file ACEL-21-e13577-s001.docx]

**EXPERIMENTAL PROCEDURES**

**Mouse strains**

In the present study, 4 factor mice (C57/B6) (Stock#011001) carrying OSKM polycystronic cassette and the rtTA trans-activator were used. Primers used for genotyping are listed in the key resource table. All animals were maintained in a vivarium monitored daily by Zhejiang Chinese Medical University Laboratory Animal Research Center. The vivarium was maintained on a 12:12 hour light and dark cycle, at a temperature of 24°C and a humidity level between 30–70%. Cages were placed on ventilated racks. Animals were provided with water and LabDiet 5LOD chow ad libitum.

**Cell culture and maintenance**

Caudal vertebrae were harvested from 4 factor mice under sterile conditions. Nucleus pulposus was extracted and incubated in 0.2% Type II collagenase under 37 °C for 40 min. The samples were centrifuged then collagenase was removed. NPC cells were cultured in Dulbecco’s modified Eagle medium (DMEM) supplemented with 10% fetal bovine serum (Gibco) and 100 U/ml penicillin–streptomycin. Subsequently, NPCs were incubated at 37 °C under a humid atmosphere with 5% CO_2_. The medium was replaced every 72 h.

**Surgically induced IDD model.**

The IDD model was established using 4 factor mice (8-week-old) through AF needle puncture . Animal were anesthetized using ketamine (100 mg/kg). A sagittal small skin incision was performed from Co6 to Co8 to help locate the disc position for needle insertion in the tail. Subsequently, Co6–Co7 coccygeal discs were punctured using a syringe needle. The syringe needle was inserted into Co6–Co7 disc along the vertical direction and then rotated by 180° in the axial direction and held for 10 s. A puncture was made parallel to the end-plates through the AF into the NP using a 32-G needle. The needle was inserted 1.5 mm into the disc to depressurize the nucleus. The other segments were not punctured to serve as control segment. Discs were harvested at week 4 and at week 14 post-surgery from 4 factor mice for subsequent IDD experiments. Doxycycline (1 mg/ml) (Sigma) was administered to mice through drinking water at week 2 post-surgery for the therapeutic experiment. The *in vivo* cyclic induction protocol comprised 2 days of doxycycline administration followed by 5 days of doxycycline withdrawal.

**Histology and immunochemistry studies**

Mouse caudal vertebrae were harvested and fixed using 4% paraformaldehyde for 24 h at room temperature. Further, the samples were decalcified in 10% EDTA for 1 month with replacement of 10% EDTA every week. Tissues were processed, paraffin embedded, and sectioned to 3 μm thick slices. Tissue sections were stained with Hematoxylin-Eosin or Safranin-O to explore annular fibrosus and nucleus pulposus . Histologic grading system was used to evaluate the degree of tissue degeneration .

**RNA isolation, cDNA synthesis, and qRT-PCR.**

Total RNA including miRNA was isolated from tissues or cultured cells using TRIzol Reagent (Ambion, Life Technologies, Carlsbad, CA, USA) according to the manufacturer’s instructions. RNA quantity and quality were determined using a nanodrop spectrophotometer (Thermo Scientific, Waltham, MA, USA) and Bioanalyzer (Agilent Inc., Santa Clara, CA, USA). Complementary DNAs were synthesized using oligo-dT primers and the iScript Select cDNA Synthesis Kit (Bio-Rad) for quantitative detection of mRNA levels. Real-time PCR analyses were performed using specific set of primers (Supplementary S1. Table) and iQSYBR Green Supermix (Bio-Rad). Normalization of gene expression levels was conducted using 18s level. All reactions were performed on a real-time PCR system (Applied Biosystems) and expression levels were analyzed using the comparative Ct (ΔΔCt) method (2−ΔΔCt with logarithm transformation).

**Western blotting**

Proteins were extracted using RIPA buffer in the presence of protease and phosphatase inhibitors (Beyotime). Protein concentration was estimated by the BCA assay (Thermo Scientific). For each sample, 20 g of protein were resuspended in sample buffer and electrophoresed in a pre-cast 10-15% Tris gel (BioRad). Samples were trabsferred to Polyvinylidene fluoride (PVDF) membranes using a BioRad Criterion system. Blots were then blocked using 5% non-fat milk/1x TBST for 1 hour at room temperature. Membranes were incubated overnight at 4°C with the following primary antibodies: β-actin (Abcam: ab178787) at 1:1000; sox9 (Abcam：ab185966) at 1:1000, oct4 (Abcam：ab181557) at 1:1000, sox2 (Abcam：ab79351) at 1:1000, c-Myc (Abcam: ab32072) at 1:1000, klf4 (Abcam: ab214666) at 1:1000 and bcl2 (Abcam: ab32124) at 1:1000. Membranes were then incubated with HRP-conjugated anti-Rabbit IgG or anti-Mouse IgG (Abcam) at 1:1000 for two hours at room temperature in 1x TBST. Protein signal was detected using enhanced chemiluminescence (ECL Prime Western Blotting System GE Healthcare). Protein molecular weight was determined using the protein marker. Western blot images were acquired and analyzed using the BioRad Image Lab system. Quantification of protein levels was performed using ImageJ software. β-actin signal was used to normalize for protein levels.

**DNA isolation and quantification of DNA methylation**

Genomic DNA was isolated from IVD tissue using TIANamp Genomic DNA Kit (cat.#DP304-02) according to the manufacturer’s instructions.

Global DNA methylation was quantified using the Colorimetric Methylated DNA Quantification Kit (Abcam, ab117128) following the manufacturer’s instructions. The percentage 5-methylcytosine (5-mC) content was calculated using the formula included in the protocol and was presented as fold-change relative to the content of the control.

**Immunofluorescence**

Cells were fixed with 4% PFA at room temperature (RT) for 15 min. Subsequently, cells were treated with 0.1% Triton X-100 in PBS for 15 min at RT. Cells were blocked with 5% BSA in PBS for 1 hr at 37 ℃ then incubated overnight at 4°C with the following primary antibodies：γ-H2AX (Cell Signaling: 9718s), H3K9me3 (Abcam: ab176916), H4K20me3 (Abcam: ab177190), oct3/4 (Sc-5279), sox2 (Abcam：ab79351), col2 (Abcam: ab34712), adamts5 (Abcam: ab41037). Cells were then washed thrice with PBS and incubated with the corresponding secondary antibody at RT for 1 hr. Nuclei were stained with DAPI (Cell Signaling: 4083s). Images were acquired using inverted fluorescence microscope (Thermo Fisher). The laser intensity was set to 3%–5% transmission of the maximum intensity, and the settings were optimized to avoid signal saturation for any of the lasers. Immunofluorescence staining results were quantified based on the mean fluorescence density using ImageJ software (National Institutes of Health).

**Cytoskeleton analysis**

Cells were fixed using 4% PFA at room temperature (RT) for 15 min. Further, cells were treated with 0.1% Triton-X-100 in PBS for 15 min at RT then labeled with 2U phalloidin (Thermo Fisher: R37110) for 30 min under dark conditions. Cells were washed thrice and fluorescence intensity was observed under an inverted fluorescence microscope.

**Senescence-associated β-galactosidase assay**

Cells were fixed in 4% PFA for 15 min at room temperature. Subsequently, cells were washed three times with PBS and incubated in staining solution containing 40 mM citric acid/Na phosphate buffer, 5 mM K4[Fe(CN)6] 3H2O, 5 mM K3[Fe(CN)6], 150 mM sodium chloride, 2 mM magnesium chloride and 1 mg/ml X-gal overnight at 37°C (without CO_2_). Cells were then washed thrice with PBS. Photographs of cells were obtained by bright field microscopy.

**Determination of reactive oxygen species levels**

Cells were washed twice with phenol red-free PBS. Cells were then incubated with 5μM DHE (Sigma D7008) in PBS for 30 min then washed twice with PBS. Cells were then incubated in an incubator for 1 h at 37 °C. Fluorescence intensity was determined using a microplate reader using Ex/Em 480/576 for detection of DHE. ROS levels were quantified based on the mean fluorescence density using ImageJ software.

**RNA sequencing**

RNA was isolated from three experimental replicates per group with 200,000 cells per sample. The samples were then submitted to lianchuan biological company for RNA sequencing analysis.

**Determination of cellular metabolism by seahorse method**

Primary nucleus pulposus cells were plated in Seahorse XF96 plates at 20,000 cells per well. Cells were then treated with or without doxycycline (2 ug/mL). Cells were serum starved for 1 h in glucose-free media containing treatments for glycolysis stress test. ECAR determination was performed prior to and after sequential addition of glucose, oligomycin and 2-DG with analyses performed every 5 min. Cells were incubated with glucose-containing media containing the treatments for 1 h for MitoStress test. Analyses were performed every 5 min prior to and after sequential addition of oligomycin, FCCP and Rotenone/Antimycin A. Cells were incubated in glucose-containing media containing treatments for 1 h for ATP rating test. Analyses were performed every 5 min prior to and after sequential addition of oligomycin and Rotenone/Antimycin A. Data were analyzed using Wave software.

**Statistical analysis**

Data were presented as mean ± SEM of least three independent experiments. Details on statistical analysis including statistical significance, and n value are reported in the Figure legends. For *in vivo* experiments n = number of animals. Statistical analyses were performed using Prism 6 Software (GraphPad). One-way ANOVA was performed for comparison of multiple group followed by Bonferroni correction. T test was conducted for statistical comparison of two group. P < 0.05 denoted statistical significance.
